# Peer Support and Community Interventions Targeting Breastfeeding in the UK: Systematic Review of Qualitative Evidence to Identify Inequities in Participants' Experiences

**DOI:** 10.1111/mcn.70041

**Published:** 2025-05-19

**Authors:** Rhiannon Evans, Caitlyn Donaldson, Rabeea'h Aslam, Joelle Kirby, Sophie Robinson, Joanne Clarke, Stephanie J. Hanley, Siang Ing Lee, Joht Singh Chandan, Ruth Garside, Jo Thompson‐Coon, Kate Jolly, Kath Maguire, Sean Harrison, G. J. Melendez‐Torres

**Affiliations:** ^1^ DECIPHer, School of Social Sciences Cardiff University Cardiff South Glamorgan UK; ^2^ Faculty of Health and Life Sciences University of Exeter Exeter Devon UK; ^3^ Institute of Applied Health Research University of Birmingham Birmingham West Midlands UK

**Keywords:** breastfeeding, community, inequality, inequity, peer support, qualitative, systematic review

## Abstract

Rates of breastfeeding remain low in the UK, with variations between population groups. Peer support and community interventions are intended to increase breastfeeding, but there is limited understanding if they cause inequities in participants' experiences. We conducted a systematic review synthesising qualitative evidence from the UK to understand: (1) what social characteristics are relevant to participants' experiences of interventions? and (2) how are participants' experiences influenced by different social characteristics? The scope of the review was informed through stakeholder consultation with women (*n* = 7) and peer supporters (*n* = 6). Searches of nine databases updated an existing systematic review. We screened relevant systematic reviews and undertook citation tracking. We conducted framework synthesis and assessed certainty of evidence with GRADE‐CERQual. Fifty‐five studies, with 68 linked reports, were included. Inequity generating experiences were identified across the course of intervention participation: (1) lack of information about intervention eligibility and culturally appropriate recruitment procedures; (2) limited accessible provision for continued attendance; (3) inadequate consideration of participation needs related to socioeconomic status, socio‐cultural background, physical characteristics, and individuals' breastfeeding journeys; and (4) enduring structural barriers (e.g. community norms) to breastfeeding inhibiting sustained behaviour post‐intervention. Evidence suggests that differential intervention experiences may lead to inequities in outcomes, particularly among individuals from different socioeconomic and ethnic backgrounds. Peer and community provision needs to be tailored to the social characteristics of different populations. Future qualitative research needs to move beyond participants' general intervention experiences and consider specific issues pertaining to recruitment, drop‐out and post‐intervention behavioural maintenance.

**Systematic Reveiw Registration:** PROSPERO CRD42024537108.

## Summary

- Breastfeeding rates in the UK are low. Peer and community interventions are intended to increase initiation and maintenance.
- Differences in experiences among population groups might lead to inequities in breastfeeding outcomes.
- Drawing together qualitative evidence from the UK, in this systematic review we identified that intervention participants, largely from lower socioeconomic and black and minority ethnic backgrounds, may have poorer experiences.
- Peer and community interventions often lack clear information on who can access them, are delivered at inaccessible times or locations, and are not tailored to the specific needs of different groups.
- Enduring structural barriers, such as cultural norms that discourage breastfeeding, can prevent post‐intervention behavioural maintenance.

## Graphical abstract

Systematic review of qualitative evidence from the UK exploring how peer support and community breastfeeding interventions might lead to inequities. There are differential experiences between population groups, particularly among individuals from different socioeconomic and socio‐cultural backgrounds.

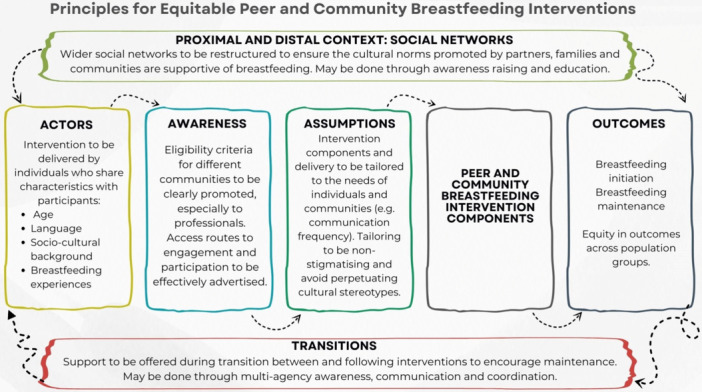

## 1 Introduction

Breastfeeding and the provision of human milk offers an accessible and cost‐effective practice that is health promoting for both the mother and the child. The World Health Organisation (WHO) recommends exclusive breastfeeding for the first six months of life, with continued breastfeeding up to two years and beyond (World Health Organisation 2003). While definitions of breastfeeding are complex and evolving (Gribble et al. 2022), it is increasingly recognised as a practice undertaken by women and birthing people, and closely relates to chest feeding, which entails the same physiological process.

Despite public health benefits, low rates of breastfeeding remain a pertinent issue in the UK, with clear inequities between population groups. Individuals who are younger, have lower levels of educational achievement, and are from lower socioeconomic and rural communities are least likely to use this feeding type (HSC Public Health Agency 2022; NHS Digital 2012; Office for Health Improvement and Disparities 2024; StatsWales 2023). Breastfeeding obstacles are notably prevalent amongst socio‐economically deprived communities, who may be exposed to unsupportive socio‐cultural infant‐feeding norms and a lack of local healthcare provision (Peregrino et al. 2018; Pérez‐Escamilla et al. 2023). Enduring inequities are considered a major social injustice, with barriers to implementing the right to breastfeed being seen as a violation of human rights (Vilar‐Compte et al. 2022).

A range of interventions have been developed to improve breastfeeding initiation and maintenance. Peer support, which provides support and counselling from trained women to individuals with similar socio‐cultural backgrounds (Jolly et al. 2012), is a common approach (Emmott et al. 2020) and recommended by National Institute for Health and Care Excellence guidelines (National Institute for Health and Care Excellence 2021). Peer support can be delivered through a variety of mechanisms and settings, including the community and hospital. It can comprise a range of different supports, notably informational support that confers knowledge and advice, appraisal support that offers encouragement and motivation, and social support that provides care, reflection and reassurance (Dennis 2003). Additional community support provision involves delivery by nonhospital‐based healthcare professionals (e.g. community health visitors) and non‐healthcare professionals. Evidence syntheses indicate mixed effectiveness for this type of support in the UK (National Institute for Health and Care Excellence 2021), partly due to low intensity of provision (Jolly et al. 2012). Meanwhile, international evidence reports issues around lack of awareness and access (Chang et al. 2022).

To date, the evidence‐base for these approaches has been limited by minimal systematic consideration of whether they generate, exacerbate, maintain or mitigate inequities between population groups, which may result from differences in participation experiences (Bengough et al. 2022; Chang et al. 2022; Leeming et al. 2022; Rojas‐García et al. 2023). However, there is an emerging, if fractured, body of primary qualitative research with a strong equity focus in the UK, which could benefit from an up‐to‐date evidence synthesis. For example, the Assets‐based feeding help Before and After birth (ABA) feasibility trial has informed a taxonomy on how breastfeeding experiences can be determined by a constellation of socio‐demographic and service‐level differences (Thomson et al. 2022).

In the present review we systematically synthesised evidence from qualitative studies and process evaluations of peer support and community breastfeeding interventions in the UK to address the following questions:

1. *What* social characteristics are identified as relevant to participants' experiences and/or views of peer support and community breastfeeding interventions?
2. *How* are participants' experiences and/or views of peer support and community breastfeeding interventions influenced by different social characteristics?

To support the review, we a priori classified social characteristics according to the Cochrane PROGRESS‐Plus framework (O'Neill et al. 2014), which supports reviews to identify characteristics that stratify health opportunities and outcomes.

We conceptualised participants' ‘experiences’ as a multidimensional construct that mapped onto discrete yet interacting phases of intervention participation (Carroll et al. 2007; Moore et al. 2015): (1) Reach and recruitment; (2) Retention; (3) Interaction; and (4) Sustainment (Table 1). This was useful in recognising that participants may have complex and contrasting experiences at different time‐points.

**Table 1:** Definition of phases of intervention experience.

Phases of intervention experience	Description
Reach and recruitment	Experience of initial engagement with the intervention and recruitment to participation. For individuals who have not participated, this may include experience of not being adequately or appropriated reached or recruited
Retention	Experiences that motivate continued engagement or encourage mid‐course withdrawal. Withdrawal may be initiated by the individual participant, delivery agents or wider contextual circumstances
Interaction	Experience of interacting with intervention mechanisms and components
Sustainment	Experience of continued engagement with intervention mechanisms and resources (e.g. online platforms or handbooks) post‐intervention delivery. These continued mechanisms and resources may be intended to sustain outcomes. For some evaluation studies, this may denote the period between posttest and longer‐term follow‐up

We note that intervention experiences are commonly described as ‘acceptability’. However, process evaluation guidance critiques the static nature of the concept, suggesting that *interaction* better reflects the dynamic relationship of participants with an intervention, and how this can change through expressions of agency (Moore et al. 2015). As such, we have indicated the phase of *participating* in an intervention as one of *interaction*.

**Figure 1:**
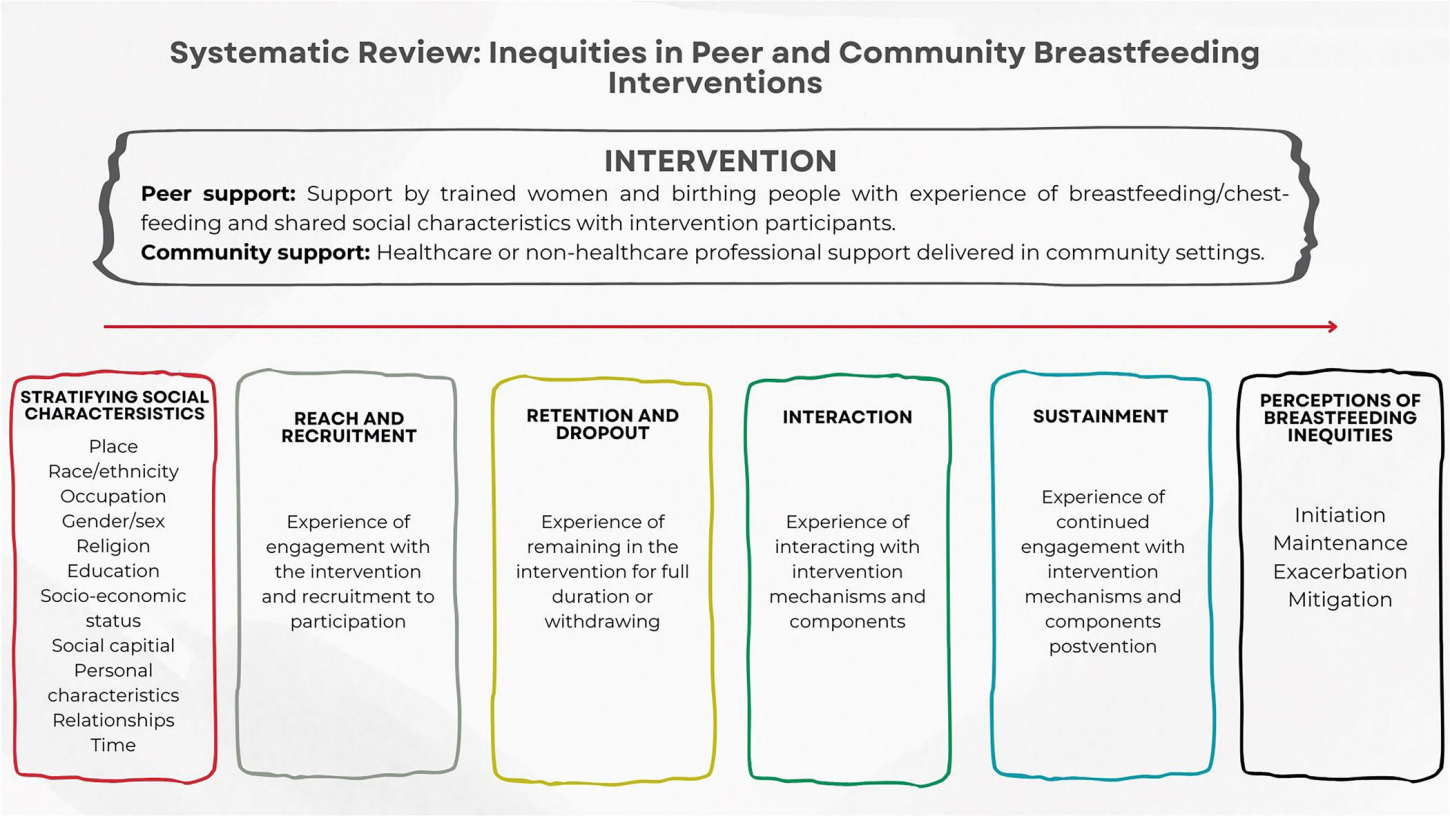
Logic model reporting parameters of systematic review.

The parameters of the review are presented in the logic model (Figure 1).

## 2 Methods

We conducted a qualitative evidence review using Framework Synthesis. We adhered to best practice in qualitive review methodology (Flemming et al. 2018) and report the review in accordance with the Enhancing transparency in reporting the synthesis of qualitative research (ENTREQ) statement (Tong et al. 2012) (Supporting Information S1).

### 2.1 Stakeholder Engagement

We conducted stakeholder engagement at two time‐points (April 2024, June 2024) with women who have breastfed (*n* = 7) and peer supporters (*n* = 6). First, we confirmed the review scope, and identified relevant social characteristics beyond those captured by PROGRESS‐Plus (O'Neill et al. 2014). Second, stakeholders provided feedback on the emergent synthesis. Stakeholders were recruited through the ABA‐feed and Multimorbidity and Pregnancy: Determinants, Clusters, Consequences and Trajectories (MuM‐PreDiCT) study.

### 2.2 Searching and Data Sources

We conducted an initial scoping search of Epistemonikos to identify existing systematic reviews that could inform the scope of the present review. From here we updated and adapted searches carried out in an existing identified review (Bengough et al. 2022), which offered a comprehensive exploration of qualitative evidence reporting women's engagement with breastfeeding strategies but did not have an equity lens. Searches were limited to December 2017 onwards, which was when searches in the Bengough et al. 2022 review were last undertaken. We conducted searches from 18th to 20th March 2024 in: Medline (Ovid); Embase (Ovid); PsycINFO (Ovid); CINAHL (EBSCO); BNI (ProQuest); Scopus (Elsevier); ASSIA (ProQuest); and Social Policy and Practice (Ovid). We also screened the references from six systematic reviews before November 2017, which were identified in the initial scoping search, and undertook backward and forward citation tracking with included study reports from Scopus (Elsevier).

### 2.3 Search Strategy

The strategy for the updated search included terms for the review's population and intervention, along with database‐appropriate subject terms, keywords and combinations. Terms for qualitative research and country were drawn from existing search filters (ISSG 2024). The search strategy was developed in Medline (Ovid) and adapted to the functionality of each bibliographic database (Supporting Information S2).

### 2.4 Inclusion Criteria

We developed an inclusion criteria tool in accordance with SPIDER (Supporting Information S3). It was refined and confirmed with stakeholders. A subsample of 100 title and abstract retrievals from bibliographic databases were screened independently and in duplicate by the review team members to calibrate the tool.

### 2.5 Study Screening Methods

Retrieved study reports were exported to Endnote 20 for de‐duplication then uploaded to Covidence for screening and extraction. Titles and abstracts were screened independently and in duplicate by two reviewers. Study reports that were included by at least one reviewer progressed to the next stage. Full texts of study reports were also independently screened by two reviewers. Conflicts in assessments of full‐texts were resolved through discussion and recourse to a third reviewer.

### 2.6 Quality Appraisal

We used a qualitative method appraisal tool developed by Wallace et al. (2004) and adapted in a subsequent public health synthesis (Shaw et al. 2023). Appraisal was conducted independently by two reviewers. A final appraisal assessment was reached through discussion (Table 2).

**Table 2:** Quality appraisal of included studies.

Studies (Including all study reports linked to studies)	1. Is the research question(s) clear?	2. Is the theoretical perspective explicit?	3. Has the theoretical perspective influenced the study design, methods and findings?	4. Is the study design appropriate to the research question(s)?	5. Is the setting adequately described?	6. Is the sample drawn from an appropriate population?	7. Are the social characteristics of the sample clearly described?	8. Is data collection adequately described?	9. Is data collection rigorous to ensure confidence in findings?	10. Is data analysis rigorous to ensure confidence in findings?	11. Are the findings presented substantiated by the data?	12. Is consideration given to limitations that may affect the results?	13. Do any claims to generalisability follow from the data?	14. Is ethics addressed and confidentiality respected?
Aiken and Thomson (2013)	Y	Y	N	Y	Y	Y	N	Y	Y	Y	Y	Y	NA	Y
Battersby (2001)	N	N	CT	CT	Y	Y	N	N	N	N	N	N	NA	CT
Beake et al. (2005)	Y	N	N	Y	Y	Y	N	Y	Y	Y	Y	N	NA	Y
Black et al. (2020)	Y	Y	Y	Y	Y	Y	N	Y	N	Y	Y	Y	N	Y
Brown and Tennant‐Eyles (2021)	Y	N	N	Y	N	Y	N	Y	N	N	Y	Y	Y	Y
Brown (2021)	Y	N	N	Y	Y	Y	Y	Y	Y	Y	Y	Y	NA	Y
Carrington‐Windo (2018)	Y	Y	Y	Y	Y	Y	N	Y	Y	Y	Y	Y	Y	Y
Cartwright and Boath (2018)	Y	Y	Y	Y	N	Y	Y	Y	N	Y	Y	Y	N	Y
Clarke et al. (2020), Ingram et al. (2020),Thomson et al. (2022), Knox et al. (2023)	Y	Y	Y	Y	Y	Y	Y	Y	Y	Y	Y	Y	Y	Y
Condon and Salmon (2015)	Y	N	CT	Y	Y	Y	Y	Y	Y	Y	Y	Y	Y	Y
Cook et al. (2021)	Y	N	N	Y	Y	Y	Y	Y	Y	Y	Y	Y	Y	Y
Copeland et al. (2019), Phillips et al. (2018), Paranjothy et al. (2017)	Y	Y	Y	Y	Y	Y	Y	Y	Y	Y	Y	Y	NA	Y
Crossland et al. (2019, 2020)	Y	Y	Y	Y	Y	Y	Y	Y	Y	Y	Y	Y	Y	Y
Dombrowski et al. (2020)	Y	Y	N	Y	Y	Y	Y	N	N	N	Y	Y	N	Y
Dowling and Pontin (2017)	N	Y	Y	Y	Y	Y	N	Y	Y	N	Y	N	NA	Y
Fox et al. (2015)	Y	N	CT	Y	Y	Y	Y	Y	Y	Y	Y	Y	NA	Y
Fraser et al. (2020)	N	N	N	Y	N	Y	Y	N	N	N	Y	N	Y	Y
Gallagher (2023)	Y	Y	Y	Y	Y	Y	Y	Y	Y	Y	Y	Y	Y	Y
Graffy and Taylor (2005)	Y	N	CT	Y	CT	Y	Y	Y	Y	Y	Y	Y	Y	CT
Hoddinott et al. (2010)	Y	N	CT	Y	Y	Y	Y	Y	Y	Y	Y	N	NA	Y
Hoddinott et al. (2010)	Y	N	N	Y	Y	Y	Y	Y	Y	Y	Y	Y	Y	Y
Hunt (2019)	Y	Y	Y	Y	Y	Y	Y	Y	Y	Y	Y	Y	Y	CT
Hunt et al. (2021)	Y	Y	Y	Y	Y	Y	Y	Y	Y	Y	Y	Y	NA	Y
Ingram et al. (2003)	Y	N	CT	Y	Y	Y	N	Y	Y	CT	Y	N	NA	CT
Ingram (2013)	Y	N	N	Y	Y	Y	Y	Y	Y	Y	Y	Y	Y	CT
Ingram (2013)	Y	N	CT	Y	Y	Y	N	Y	Y	Y	Y	Y	NA	Y
Islam (2016)	Y	N	CT	Y	Y	Y	Y	Y	Y	CT	Y	Y	Y	CT
Jackson and Hallam (2019, (2021)	Y	CT	CT	Y	Y	Y	N	Y	Y	CT	Y	Y	Y	N
Jackson et al. (2024)	Y	Y	Y	Y	Y	Y	N	Y	Y	Y	Y	Y	NA	Y
Jamie et al. (2020)	Y	Y	Y	Y	N	Y	Y	Y	CT	Y	Y	Y	NA	Y
Johnson et al. (2018)	Y	Y	CT	Y	Y	Y	Y	Y	Y	Y	Y	Y	N	Y
Lyons et al. (2019)	Y	Y	Y	Y	N	Y	N	Y	Y	Y	Y	Y	N	Y
Marshall et al. (2007)	Y	N	N	Y	N	Y	Y	Y	Y	Y	Y	N	N	Y
McFadden and Toole (2006)	Y	N	CT	Y	Y	Y	N	Y	Y	Y	Y	Y	NA	Y
McFadden et al. (2013)	Y	N	N	Y	Y	Y	Y	Y	Y	Y	Y	Y	Y	Y
Mengoni et al. (2023)	Y	Y	Y	Y	N	Y	Y	Y	Y	Y	Y	Y	Y	Y
Miller (2022)	Y	CT	CT	Y	Y	Y	CT	Y	Y	Y	Y	Y	N	Y
Morse and Brown (2021), Morse and Brown (2021), Morse and Brown (2021)	Y	Y	Y	Y	Y	Y	Y	Y	Y	Y	Y	Y	Y	N
Morse and Brown (2021)	Y	Y	Y	Y	N	Y	N	Y	N	N	Y	Y	NA	Y
Newman and Williamson (2018)	Y	Y	Y	Y	Y	Y	Y	Y	Y	Y	Y	Y	Y	Y
Psarros (2018)	Y	N	CT	Y	Y	Y	Y	Y	Y	CT	Y	Y	Y	CT
Raine (2003)	Y	Y	Y	Y	N	Y	N	Y	Y	Y	Y	N	N	CT
Regan and Brown (2019)	Y	N	N	Y	Y	Y	N	N	Y	Y	Y	Y	Y	CT
Scott et al. (2003)	Y	N	CT	Y	Y	Y	Y	Y	Y	Y	Y	N	NA	N
Simmons (2021)	Y	Y	Y	Y	Y	Y	Y	Y	Y	Y	Y	Y	Y	Y
Smyth (2020)	Y	Y	Y	Y	Y	Y	Y	N	N	N	Y	N	NA	N
Tan et al. (2017)	Y	N	N	Y	Y	Y	Y	Y	Y	N	Y	Y	N	N
Thompson et al. (2020)	Y	Y	Y	Y	Y	Y	Y	Y	Y	Y	Y	Y	Y	Y
Thomson et al. (2012)	Y	N	N	Y	Y	Y	N	Y	Y	Y	Y	Y	N	Y
Thomson et al. (2012, 2015)	Y	Y	Y	Y	Y	Y	Y	Y	Y	Y	Y	Y	Y	Y
Thomson and Crossland (2013)	Y	N	N	Y	Y	Y	Y	Y	Y	Y	Y	Y	Y	Y
Thomson et al. (2015)	Y	Y	Y	Y	Y	Y	Y	Y	N	Y	Y	N	N	Y
Trickey (2018)	Y	Y	Y	Y	Y	Y	Y	Y	Y	Y	Y	Y	Y	Y
Wade et al. (2009)	Y	Y	Y	Y	Y	Y	N	Y	N	Y	Y	N	N	Y
Wagg et al. (2021, 2022)	Y	Y	Y	Y	Y	Y	N	Y	N	N	Y	N	NA	Y

*Note:* Appraised quality of studies and related study reports. Appraisal conducted according to the Wallace et al. (2004) appraisal tool.

Abbreviations: CT = cannot tell, N = no, NA = not applicable, Y = yes.

### 2.7 Data Extraction and Coding

Data extraction was guided by an a priori framework that mapped onto the review questions and pre‐specified phase of intervention: author; publication year; title; aim; country; setting; data collection year; intervention characteristics; intervention partcipation phase; PROGRESS‐Plus or other equity characteristics; participants generating data; data collection method; and data. For the data constructs that related to participants' experiences we conducted inductive line‐by‐line coding. Coding was conducted by one member of the review team and checked by another.

To support the order of study reports entering the synthesis, we classified them as: (1) High equity relevance: Reports that directly engaged with target populations and addressed inequities; (2) Medium equity relevance: Reports focused on inequities but generated data with stakeholders that are not the target participants; and (3) Low equity relevance: Reports that did not explicitly consider inequities.

### 2.8 Synthesis

To address research question one, we charted the social characteristics assessed in each study report and cross‐referenced these with stakeholder priority characteristics to assess if the evidence‐base is assessing real‐world intervention needs. For research question two, we grouped studies by intervention experience phase, and then generated themes (e.g. cultural awareness) from inductively coded data within each phase. Recommendations for future practice initially ran across phases but to strengthen their presentation we placed them into a separate category, which is included in the discussion. We entered high equity relevance studies into the synthesis first. Themes were developed by the wider review team, and further refined through stakeholder consultation.

### 2.9 Synthesis Output

Frequencies of reported social characteristics are presented in a summary table. The synthesis is presented as a narrative summary with summary tables. We generated an infographic to illustrate principles for equitable interventions. For the purposes of the synthesis, we have used the language originally presented in the primary studies.

### 2.10 Certainty of Evidence

We used the GRADE‐CERQual tool to assess the certainty of evidence (Lewin et al. 2018; Lewin et al. 2015). We generated an Evidence Profile and Summary of Qualitative Findings (SoQF) table.

## 3 Results

### 3.1 Study Characteristics

**Figure 2:**
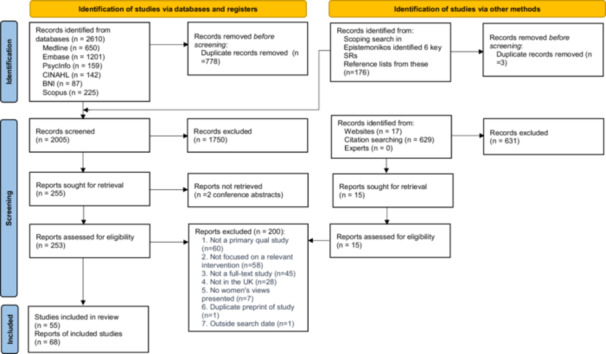
PRISMA 2020 flow diagram for new systematic reviews which included searches of databases, registers and other sources. *Source* (PRISMA diagram template)*:* Page MJ, et al. BMJ 2021;372:n71. doi: 10.1136/bmj.n71.

A total of 1832 unique study reports were identified. Fifty‐five studies, with 68 linked study reports, were included (Figure 2).

Of the included studies, 35 were conducted in England, two in Northern Ireland, four in Scotland and two in Wales. Eight were conducted across the UK, two delivered in England and Wales only, one in England and Northern Ireland, and one in England, Wales, and Scotland. Twenty‐three studies focused on peer support, 19 examined community provision, and 13 considered both. Seventeen studies reported on the intervention phase of reach and recruitment, four on retention, 54 on interaction, and seven on sustainment. We classified 34 studies as having high equity relevance, two as having medium equity relevance and 19 as having low equity relevance.

### 3.2 Quality Appraisal

While studies varied in methodological rigour, most were rated high quality (Table 2). Strengths included clear articulation of research questions and use of an appropriate design. Common limitations were lack of a guiding theoretical perspective and its application. Only a few studies clearly described equity relevant social characteristics. As the review had a specific UK focus we experienced challenges in assessing generalisability. As such, we frequently assessed this domain as not applicable.

### 3.3 Social Characteristics Relevant to Participants' Experiences

We identified potential inequity generating social characteristics through PROGRESS‐Plus and shared these with stakeholders. They confirmed all domains were relevant and recommended further characteristics that were rarely supported by intervention: relationship to partner; mixed (i.e., combination of breast and bottle‐feeding) or bottle‐feeding; negative prior breastfeeding experiences; larger bodies; neurodiversity; and darker skin tones.

The frequency of studies exploring each social characteristic is presented in Table 3. Characteristics were only reported in high and medium equity studies, and each study could report multiple characteristics. The most common characteristics were socio‐demography and race/ethnicity/culture and language. Of the characteristics prioritised by stakeholders, no studies explicitly addressed: relationship to partner; neurodiversity; and skin tone. We did not identify social characteristics from studies that were not previously identified from PROGRESS‐Plus or stakeholder consultation.

**Table 3:** Frequency of social characteristics reported by studies.

Equity characteristic	No. of studies
*PROGRESS‐Plus Characteristics*	
Place of residence	1
Race/ethnicity/culture/language	10
Occupation	—
Gender/sex	1
Religion	1
Education	—
Socioeconomic status	18
Social capital	1
Personal characteristics (Child disability)	3
Personal characteristics (Age)	4
Features of relationships (Relationship to partner)	—
Time‐dependent relationships (Short vs. Longer‐term breastfeeding)	6
*Additional stakeholder consultation priority characteristics*	
Feeding type (Breast vs. Mixed vs. Bottle)	3
Previous experience of breastfeeding	1
Body type	1
Neurodiversity	—
Skin tone	—

### 3.4 Phases of Intervention Experience

The synthesis is reported according to the four phases of intervention experience (Tables 4 and 5). The study reports linked to each intervention phase are reported in Supporting Information S4.

**Table 4:** Overview of study characteristics.

Study	Equity relevance	Intervention type	Targeted social characteristics	Targeted outcomes	Phase of intervention experience	Method	Sample
Battersby (2001)	High	Peer support	Socioeconomic status	Breastfeeding/chest feeding initiation; Breastfeeding/chest feeding maintenance; Breastfeeding/chest feeding (not specified)	Interaction	Interview; Mixed method	Mothers: *n* = 5 (interviewed); 16 (completed audit questionnaire) Professionals/Peer Supporters: *n* = Not specified Age of Mother: Not specified Socioeconomic Status: Not specified Ethnicity: Not specified
Beake et al. (2005)	High	Community	Socioeconomic status	Breastfeeding/chest feeding initiation; Breastfeeding/chest feeding maintenance	Reach and recruitment; Interaction	Interview; Other	Mothers: *n* = Not specified Professionals/Peer Supporters: *n* = Not specified Age of Mother: Not specified Socioeconomic Status: High‐deprivation area with indicators such as high levels of temporary accommodation, high number of refugees, teenage pregnancy, low literacy, and numeracy rates. Ethnicity: Not specified
Brown and Tennant‐Eyles (2021)	High	Community	Race/ethnicity/culture; Mixed feeding	Breastfeeding/chest feeding initiation; Breastfeeding/chest feeding maintenance	Reach and recruitment; Interaction; Sustainment	Interview; Mixed method	Mothers: *n* = 70 Professionals/Peer Supporters: *n* = Not specified Age of Mothers: Not specified Socioeconomic Status: Various (low‐income, rurality) Ethnicity: Not specified
Brown and Tennant‐Eyles (2021)	Medium	Peer support	Place; Race/ethnicity/culture; Socioeconomic status; Disability	Breastfeeding/chest feeding (not specified)	Reach and recruitment; Retention; Interaction;	Interview; Focus group	Mothers: *n* = 81 Professionals/Peer Supporters: *n* = 20 Age of Mothers: Not specified Socioeconomic Status: Various (asylum seekers, Gypsy, and Traveller) Ethnicity: Various (asylum seekers, Gypsy, and Traveller)
Cartwright and Boath (2018)	High	Community	Disability	Breastfeeding/chest feeding initiation; Breastfeeding/chest feeding (not specified)	Interaction	Focus group	Mothers: *n* = 8 Professionals/Peer Supporters: *n* = Not specified Age of Mother: 29–44 years Socioeconomic Status: Not specified Ethnicity: All White British
Clarke et al. (2020), Ingram et al. (2020), Thomson et al. (2022), Knox et al. (2023)	High	Peer support	Socioeconomic status	Breastfeeding/chest feeding initiation; Breastfeeding/chest feeding maintenance	Reach and recruitment; Retention; Interaction	Interview; Focus group; Process evaluation (specifies embedded in RCT or other outcome evaluation); Other	Mothers: *n* = 103 (Intervention Group); *n* = 30 (Qualitative Interviews) Professionals/Peer Supporters: *n* = 17 (Infant feeding helpers, IFH manager, healthcare providers) Age of Mother: Average 28.5 years (Range: 17.7–43.0) Socioeconomic Status: Deprivation Index: –24.5% from IMD 1 (most deprived) –12.8% from IMD 2 –18.6% from IMD 3%–26.5% from IMD 4 –17.7% from IMD 5 (least deprived) Employment: 88.2% in paid work Education: 45.1% had a degree or higher Ethnicity: 86.3% White British (Intervention Group); 86.6% White British (Qualitative Interviews)
Condon and Salmon (2015)	High	Community	Race/ethnicity/culture	Breastfeeding/chest feeding (not specified)	Interaction	Interview	Mothers: *n* = 22 Professionals/Peer Supporters: *n* = Not specified Age of Mothers: Not specified Socioeconomic Status: Not specified Ethnicity: Half Roma, Gypsy, and Irish Traveller backgrounds
Cook et al. (2021)	High	Community	Race/ethnicity/culture	Breastfeeding/chest feeding (not specified)	Reach and recruitment; Interaction	Focus group	Mothers: *n* = 63 Professionals/Peer Supporters: *n* = Not specified Age of Mothers: 21–45 years Socioeconomic Status: Not specified Ethnicity: White British, South Asian, Black, Polish
Copeland et al. (2019), Phillips et al. (2018), Paranjothy et al. (2017)	High	Peer support	Socioeconomic status	Breastfeeding/chestfeeding maintenance; Breastfeeding/chest feeding (not specified)	Interaction; Sustainment	Interview; Participant observation; Diaries; Process evaluation	Mothers: *n* = 29 Professionals/Peer Supporters: *n* = 20 Age of Mothers: Not specified Socioeconomic Status: Not specified Ethnicity: Not specified
Dombrowski et al. (2020)	High	Community	Socioeconomic status	Breastfeeding/chest feeding maintenance	Interaction	Focus group	Mothers: *n* = Not specified Professionals/Peer Supporters: *n* = Not specified Age of Mothers: Over 18 years Socioeconomic Status: Not specified Ethnicity: Not specified
Dowling and Pontin (2017)	High	Peer support	Longer term breastfeeding	Breastfeeding/chest feeding maintenance	Interaction	Interview; Ethnography; Participant observation; Other	Mothers: *n* = 6 (interviews); 80+ (observations) Professionals/Peer Supporters: *n* = Not specified Age of Mother: 27–42 years Socioeconomic Status: Not specified Ethnicity: Not specified
Gallagher (2023)	High	Peer support; Community	Socioeconomic status	Breastfeeding/chest feeding initiation; Breastfeeding/chest feeding (not specified)	Interaction	Interview	Mothers: *n* = 9 Professionals/Peer Supporters: *n* = Not specified Age of Mothers: Not specified Socioeconomic Status: Not specified Ethnicity: Not specified
Hoddinott et al. (2010)	High	Community	Socioeconomic status	Breastfeeding/chest feeding (not specified)	Interaction	Interview	Mothers *n* = 36 Professionals/Peer Supporters: *n* = 37 (Significant Others), 26 (Partners), 8 (Maternal Mothers), 1 (Sister), 2 (Health Professionals) Age of Mother: Not specified Socioeconomic Status: A high proportion from the most deprived quintiles according to the Scottish Index of Multiple Deprivation Ethnicity: Not specified
Hunt (2019)	High	Peer support	Socioeconomic status	Breastfeeding/chest feeding maintenance	Interaction	Interview; Focus group	Mothers: *n* = Not specified Professionals/Peer Supporters: *n* = Not specified Age of Mothers: 20–40+ years Socioeconomic Status: Deprived postcodes Ethnicity: Not specified
Hunt et al. (2021), Hunt (2019)	High	Peer support	Socioeconomic status	Breastfeeding/chest feeding initiation; Breastfeeding/chest feeding maintenance; Breastfeeding/chest feeding (not specified)	Reach and recruitment; Sustainment; Interaction	Interview; Observation	Mothers: *n* = Not specified Professionals/Peer Supporters: *n* = Not specified Age of Mothers: 23–37 years Socioeconomic Status: Varied (IMD quintiles 1–4) Ethnicity: Mostly White British
Ingram (2013)	High	Peer support; community	Race/ethnicity/culture; Age	Breastfeeding/chest feeding (not specified)	Interaction	Focus group	Mothers: *n* = 22 Professionals/Peer Supporters: *n* = Not specified Age of Mothers: Not specified Socioeconomic Status: Not specified Ethnicity: Somali, Afro‐Caribbean, South Asian
Ingram (2013)	High	Peer support	Socioeconomic status	Breastfeeding/chest feeding initiation	Interaction; Sustainment	Interview; Focus group; Other	Mothers: *n* = 163 Professionals/Peer Supporters: *n* = 12 Age of Mothers: Mean 29.6 years Socioeconomic Status: Not specified Ethnicity: Not specified
Islam (2016)	High	Peer support	Socioeconomic status	Breastfeeding/chest feeding initiation; Breastfeeding/chest feeding maintenance; Breastfeeding/chest feeding (not specified)	Reach and recruitment	Interview	Mothers: *n* = Not specified Professionals/Peer Supporters: *n* = Not specified Age of Mothers: Mean 29 years Socioeconomic Status: Low income Ethnicity: All White British
Jackson and Hallam (2019, 2021)	High	Peer support; community	Longer term breastfeeding	Breastfeeding/chest feeding initiation; Breastfeeding/chest feeding maintenance	Reach and recruitment; Retention Interaction; Sustainment	Interview	Mothers: *n* = 24 Professionals/Peer Supporters: *n* = Not specified Age of Mother: 27–48 years Socioeconomic Status: Varied (58% postgraduate, 21% undergraduate, 17% college, 4% high school) Ethnicity: Predominantly White British
Jackson et al. (2024)	High	Community	Gender/sex	Breastfeeding/chest feeding (not specified)	Interaction	Interview	Mothers: *n* = 10 Professionals/Peer Supporters: *n* = Not specified Age of Mother: Mean 33 years Socioeconomic Status: Not specified Ethnicity: Not specified
Jamie et al. (2020)	High	Community	Age	Breastfeeding/chest feeding initiation	Interaction	Interview; Focus group; Creative methods	Mothers: *n* = 27 Professionals/Peer Supporters: *n* = Not specified Age of Mother: 16–24 years Socioeconomic Status: Not specified Ethnicity: 26 White British, 1 Mixed race
Lyons et al. (2019)	High	Peer support	Socioeconomic status; Body type	Breastfeeding/chest feeding (not specified)	Interaction	Interview	Mothers: *n* = 18 Professionals/Peer Supporters: *n* = Not specified Age of Mothers: Not specified Socioeconomic Status: BMI ≥ 30 kg/m² Ethnicity: Not specified
Marshall et al. (2007)	High	Peer support; community	Mixed feeding	Breastfeeding/chest feeding (not specified)	Interaction	Interview; Participant observation	Mothers: *n* = 22 Professionals/Peer Supporters: *n* = Not specified Age of Mothers: Not specified Socioeconomic Status: Not specified Ethnicity: Predominantly White
McFadden and Toole (2006)	High	Peer support; community	Race/ethnicity/culture; Socioeconomic status; Age	Breastfeeding/chest feeding (not specified)	Interaction	Focus group	Mothers: *n* = Not specified Professionals/Peer Supporters: *n* = Not specified Age of Mothers: Not specified Socioeconomic Status: Low‐income, minority ethnic groups Ethnicity: Various (minority ethnic groups)
McFadden et al. (2013)	High	Peer support; community	Race/ethnicity/culture; Language; Religion	Breastfeeding/chest feeding (not specified)	Reach and recruitment; Interaction	Interview; Focus group	Mothers: *n* = 23 Professionals/Peer Supporters: *n* = 32 Age of Mothers: Not specified Socioeconomic Status: Not specified Ethnicity: Bangladeshi, White British
Mengoni et al. (2023)	High	Peer support	Disability	Breastfeeding/chest feeding (not specified)	Reach and recruitment; Interaction	Interview	Mothers: *n* = 8 Professionals/Peer Supporters: *n* = 12 Age of Mother: Not specified Socioeconomic Status: Not specified Ethnicity: Not specified
Newman and Williamson (2018)	High	Community	Race/ethnicity/culture; Longer term breastfeeding	Breastfeeding/chest feeding (not specified)	Interaction	Interview	Mothers: *n* = 8 Professionals/Peer Supporters: *n* = Not specified Age of Mothers: 26–36 years Socioeconomic Status: Not specified Ethnicity: Not specified
Psarros (2018)	High	Peer support; community	Race/ethnicity/culture; Age	Breastfeeding/chest feeding (not specified)	Interaction	Focus group	Mothers: *n* = 81 Professionals/Peer Supporters: *n* = 20 Age of Mothers: Not specified Socioeconomic Status: Not specified Ethnicity: Various minority backgrounds (e.g., Gypsy, Roma, Orthodox Jewish)
Raine (2003)	High	Peer support; community	Socioeconomic status; Social capital	Breastfeeding/chest feeding (not specified)	Interaction	Interview; Participant observation; Diaries	Mothers: *n* = 6 (Breastfeeding Mothers) Professionals/Peer Supporters: *n* = 20 (Including Project Coordinator, Midwives, Health Visitors, Breastfeeding Supporters) Age of Mother: Range: 24–41 years (Average age 33 years) Socioeconomic Status: High disadvantage area with significant social and cultural barriers to breastfeeding. Ethnicity: Not specified
Scott et al. (2003)	High	Peer support	Socioeconomic status	Breastfeeding/chest feeding (not specified)	Interaction	Focus group	Mothers: *n* = 19 Professionals/Peer Supporters: *n* = Not specified Age of Mothers: Not specified Socioeconomic Status: Most socially deprived areas Ethnicity: Not specified
Thompson et al. (2020)	High	Peer support; community	Longer term breastfeeding	Breastfeeding/chest feeding (not specified)	Interaction	Interview	Mothers: *n* = Not specified Professionals/Peer Supporters: *n* = Not specified Age of Mother: At least 18 years Socioeconomic Status: Highly educated Ethnicity: Predominantly White
Thomson et al. (2012, 2015)	Medium	Peer support	Race/ethnicity/culture	Breastfeeding/chest feeding initiation; Breastfeeding/chest feeding maintenance	Reach and recruitment; Interaction sustainment	Interview	Mothers: *n* = 47 (Paper 1); 87 (Paper 2) Professionals/Peer Supporters: *n* = 50 (health and community professionals) Age of Mothers: 19–39 years Socioeconomic Status: Not specified Ethnicity: Predominantly White British/European
Thomson et al. (2012)	High	Peer support	Socioeconomic status	Breastfeeding/chest feeding initiation; Breastfeeding/chest feeding maintenance	Interaction	Interview; Focus group	Mothers: *n* = 26 Professionals/Peer Supporters: *n* = 4 (focus group) Age of Mothers: 21–42 years Socioeconomic Status: Not specified Ethnicity: 25 White‐British, 1 Asian origin
Thomson and Crossland (2013)	High	Peer support	Mixed feeding	Breastfeeding/chest feeding (not specified)	Reach and recruitment; Interaction	Interview	Mothers: *n* = 47 (Paper 1); 87 (Paper 2) Professionals/Peer Supporters: *n* = 6 (volunteer coordinators) Age of Mother: 19–39 years (Mean = 29 years) Socioeconomic Status: Not specified Ethnicity: 85.7% White
Trickey (2018)	High	Peer support	Socioeconomic status	Breastfeeding/chest feeding (not specified)	Retention; Interaction	Interview; Focus group; Creative methods	Mothers: *n* = Not specified Professionals/Peer Supporters: *n* = 15 policy participants Age of Mothers: Not specified Socioeconomic Status: Low‐income Valleys communities Ethnicity: Not specified
Wagg et al. (2021)	High	Peer support; community	Longer term breastfeeding	Breastfeeding/chest feeding (not specified)	Interaction	Interview	Mothers: *n* = 10 Professionals/Peer Supporters: *n* = Not specified Age of Mother: Not specified Socioeconomic Status: Not specified Ethnicity: Not specified

**Table 5:** Overview of synthesis results.

Study	Equity relevance	Intervention type	Outcomes	Social characteristics	Method	Summary of results
Battersby (2001)	High	Peer support	Breastfeeding/chest feeding initiation; Breastfeeding/chest feeding maintenance; Breastfeeding/chest feeding (not specified)	Socioeconomic status	Interview; Mixed method	**Interaction:** Mothers found antenatal peer support workers more effective than classes, valuing their personalised, ongoing support, which extended breastfeeding duration and highlighted the need for flexible support options and experience‐based selection criteria.
Beake et al. (2005)	High	Community	Breastfeeding/chest feeding initiation; Breastfeeding/chest feeding maintenance	Socioeconomic status	Interview; Other	**Reach and Recruitment:** Antenatal contact with women referred by midwives was prioritised, with postnatal referrals being slower. Support workers, checking birth registers, made early visits and were labelled as Infant Feeding Support Workers to remain inclusive to those initially considering bottle‐feeding. **Interaction:** Support workers focused on encouragement, practical feeding advice, confidence‐building, and referrals. Women valued their hands‐on support, information, and personal experience, highlighting the importance of advice from those who had breastfed themselves.
A. Brown (2021)	High	Community	Breastfeeding/chest feeding initiation; Breastfeeding/chest feeding maintenance	Race/ethnicity/culture; Mixed feeding	Interview; Mixed method	**Reach and Recruitment:** Mothers were asked about their experiences when they first became aware of the programme and why they decided to join. **Interaction:** Mothers reflected on how the Peppy programme supported their breastfeeding through webinars, text messages, and one‐to‐one expert consultations. Health professionals valued Peppy for its consistent support and online accessibility, especially during lockdown, contrasting it with perceived deficiencies in NHS maternity services. **Sustainment:** Support from Peppy practitioners across different feeding modes encouraged Ruth, who didn't breastfeed her first baby, to consider breastfeeding next time due to the reliable support provided.
Brown and Tennant‐Eyles (2021)	Medium	Peer support	Breastfeeding/chest feeding (not specified)	Place; Race/ethnicity/culture; Socioeconomic status; Disability	Interview; Focus groups	**Reach and recruitment:** Peer supporters struggled with access issues due to poor locations, transport, and weekend availability, compounded by inadequate signposting from health professionals and a reliance on underfunded volunteer services. **Retention:** Pandemic‐related online peer support saw initial attendance but declined due to online fatigue, as virtual formats lacked the same level of social support. **Interaction:** Peer support services often failed to represent the diverse demographics of attendees, particularly in hospital settings, and while they provided practical advice and a non‐judgmental environment, they sometimes struggled to engage the most marginalised or socio‐economically disadvantaged groups, especially in rural and deprived areas.
Cartwright and Boath (2018)	High	Community	Breastfeeding/chest feeding initiation; Breastfeeding/chest feeding (not specified)	Disability	Focus group	**Interaction:** Healthcare professionals often struggled to support mothers with breastfeeding, particularly those with babies who had additional health needs, leaving many mothers frustrated by a lack of practical solutions and guidance.
Clarke et al. (2020), Ingram et al. (2020), Thomson et al. 2022), Knox et al. (2023)	High	Peer support	Breastfeeding/chest feeding initiation; Breastfeeding/chest feeding maintenance	Socioeconomic status	Interview; Focus group	**Reach and recruitment:** Women found the recruitment process helpful and supportive, often reinforcing their decision to breastfeed, while midwives appreciated the researcher's clear explanations. However, some women faced challenges accessing support due to travel issues or misperceptions that support was only for exclusive breastfeeding. **Retention and dropout:** Geographical distance made home visits challenging for Infant Feeding Helpers, leading to travel difficulties, and impacting some women's comfort in requesting postnatal support, despite their positive feelings about the service. **Interaction:** Antenatal meetings were praised for their supportive, woman‐centred approach, while text messaging and the assets leaflet were appreciated, though travel difficulties and mixed reactions to the genogram presented challenges.
Condon and Salmon (2015)	High	Community	Breastfeeding/chest feeding (not specified)	Race/ethnicity/culture	Interview	**Interaction:** Mothers took pride in their parenting abilities, with Roma communities viewing breastfeeding as a cultural norm, while Irish Traveller/Gypsy communities faced cultural taboos and logistical challenges. Effective support often depended on building trusted relationships with health professionals, despite issues with accessing written information due to language and literacy barriers.
Cook et al. (2021)	High	Community	Breastfeeding/chest feeding (not specified)	Race/ethnicity/culture	Focus group	**Reach and recruitment:** Inequity in access was predominantly linked to language, culture, ethnicity, and socioeconomic deprivation, with a persistent lack of culturally tailored support for migrant communities. **Interaction:** Younger white British mothers were often critical of healthcare professionals, feeling judged and unsupported, while South Asian mothers highlighted a lack of help in certain areas. Polish mothers, who faced significant breastfeeding challenges, did not report accessing support, suggesting a need for culturally tailored services to better meet the needs of diverse communities.
Copeland et al. (2019), Phillips et al. (2018), Paranjothy et al. (2017)	High	Peer support	Breastfeeding/chest feeding maintenance; Breastfeeding/chest feeding (not specified)	Socioeconomic status	Interview; Participant observation; Diaries; Process evaluation	**Interaction:** Antenatal contact with peer supporters helped build trust and comfort, though some found the frequency excessive, and text messages were valued for timely support, with suggestions for electronic information to reduce paperwork confusion. **Sustainment:** Mothers suggested a more gradual transition from Mam‐Kind support to community services, preferring continued, phased follow‐up to ease the end of support.
Dombrowski et al. (2020)	High	Community	Breastfeeding/chest feeding maintenance	Socioeconomic status	Focus group	**Interaction:** Mothers felt they bore the responsibility of educating nursery staff about supporting breastfeeding, and expressed concerns about practitioners' knowledge, adherence to feeding schedules, and safe milk storage facilities.
Dowling and Pontin (2017)	High	Peer support	Breastfeeding/chest feeding maintenance	Longer term breastfeeding	Interview; Ethnography; Participant observation; Other	**Interaction:** Many women sought alternative support through specialised networks for long‐term breastfeeding and related parenting choices, finding community and belonging both in physical groups and online spaces.
Gallagher (2023)	High	Peer support; Community	Breastfeeding/chest feeding initiation; Breastfeeding/chest feeding (not specified)	Socioeconomic status	Interview	**Interaction:** Health visitors pressured mothers with inconsistent advice and a lack of praise, while socioeconomic factors led some to avoid breastfeeding groups where formula feeding was less accepted.
Hoddinott et al. (2010)	High	Community	Breastfeeding/chest feeding (not specified)	Socioeconomic status	Interview	**Interaction:** Women wanted realistic feeding advice, faced barriers to asking for help, and needed timely support from health professionals and family to continue breastfeeding and delay introducing solids.
Hunt (2019)	High	Peer support	Breastfeeding/chest feeding maintenance	Socioeconomic status	Interview; Focus group	**Interaction:** Women experienced pressure to breastfeed with inadequate support. They found group settings unhelpful and feared peer supporters might judge them like healthcare professionals, leading to reluctance in seeking help.
Hunt et al. (2021), Hunt (2019)	High	Peer support	Breastfeeding/chest feeding initiation; Breastfeeding/chest feeding maintenance; Breastfeeding/chest feeding (not specified)	Socioeconomic status	Interview; Observation	**Reach and Recruitment:** Breastfeeding support services often failed to address the specific needs of disadvantaged mothers, with recruitment and engagement strategies better suited to more advantaged groups, leading to unequal access and effectiveness of support. **Retention:** Mothers highly valued peer support for its practical, emotional, and informational benefits, appreciating the availability, non‐judgmental attitudes, and respect for their needs, though some did not engage due to personal preferences or lack of opportunity. **Interaction:** Organisations used person‐centred, non‐directive support approaches, integrating local context and experiential knowledge to build trust and address both individual and socio‐cultural challenges in breastfeeding support. **Sustainment:** One‐to‐one peer support for breastfeeding not only aided individuals but also catalysed broader community change, with embedded services, active community engagement, and cultural advocacy driving long‐term shifts in attitudes, despite challenges from service disruptions and varying levels of engagement.
Ingram (2013)	High	Peer support; community	Breastfeeding/chest feeding (not specified)	Race/ethnicity/culture; Age	5 Focus group	**Interaction:** Breastfeeding support groups were favoured for broader baby‐related and social support, with Somali and South Asian women preferring ethnic‐specific groups, Afro‐Caribbean women favoured multicultural groups, and young mothers desired peer‐specific groups.
Ingram (2013)	High	Peer support	Breastfeeding/chest feeding initiation	Socioeconomic status	Interview; Focus group; Other	**Interaction:** Both mothers and peer supporters valued antenatal visits for pre‐baby breastfeeding preparation, and mothers appreciated the convenience of text communication for post‐birth support. **Sustainment:** Women found breastfeeding groups helpful for continued support beyond the initial two weeks, with peer supporters providing valuable ongoing assistance.
Islam (2016)	High	Peer support	Breastfeeding/chest feeding initiation; Breastfeeding/chest feeding maintenance; Breastfeeding/chest feeding (not specified)	Socioeconomic status	Interview	**Reach and recruitment**: Low engagement with breastfeeding support services was attributed to factors such as lack of awareness, difficulty asking for help, reliance on other support, and concerns about peer supporter interactions and breastfeeding challenges.
Jackson and Hallam (2019, 2021)	High	Peer support; community	Breastfeeding/chest feeding initiation; Breastfeeding/chest feeding maintenance	Longer term breastfeeding	Interview	**Reach and recruitment:** Women often had to advocate for tailored breastfeeding support, which was particularly challenging for those with limited breastfeeding experience. **Retention:** Securing accessible, low‐cost venues for breastfeeding groups became increasingly challenging, particularly after funding cuts to key community provisions like Children's Centres. **Interaction:** Mothers breastfeeding beyond early infancy perceived healthcare professionals were misinformed about longer‐term breastfeeding, often turning to online communities and peer support for more relevant expertise. Online communities like “Breastfeeding Babies and Beyond” provided women with immediate, practical advice on breastfeeding, including etiquette, fertility, and feeding during pregnancy, often viewed as more dependable than traditional medical advice. **Sustainment:** La Leche League meetings provided women with a supportive community, empowering them through exposure to extended and tandem breastfeeding practices, reducing stigma, and offering practical advice and reassurance often lacking from healthcare providers.
Jackson et al. (2024)	High	Community	Breastfeeding/chest feeding (not specified)	Gender/sex	Interview	**Interaction:** Parents expressed a need for more accessible and specialised support for bodyfeeding, highlighting issues such as the lack of inclusive language, insufficient awareness of conditions like d‐MER, and the need for representation and understanding of diverse experiences in healthcare settings.
Jamie et al. (2020)	High	Community	Breastfeeding/chest feeding initiation	Age	Interview; Focus group; Creative methods	**Interaction:** Women appreciated their supportive health visitor but would have preferred for breastfeeding guidance from peers of her own age as well.
Lyons et al. (2019)	High	Peer support	Breastfeeding/chest feeding (not specified)	Socioeconomic status; Body type	Interview	**Overall experience:** Women with larger bodies found that feeling empowered and having realistic expectations about breastfeeding, supported by peers and practical advice, helped them overcome social and practical challenges.
Marshall et al. (2007)	High	Peer support; community	Breastfeeding/chest feeding (not specified)	Mixed feeding	Interview; Participant observation	**Interaction:** Challenge with how women balance the expectations of ‘good mothering’ with practical breastfeeding support, navigating conflicting advice, personal support, and self‐assessment to manage their breastfeeding experiences.
McFadden and Toole (2006)	High	Peer support; community	Breastfeeding/chest feeding (not specified)	Race/ethnicity/culture; Socioeconomic status; Age	Focus group	**Interaction:** Bangladeshi women preferred support from culturally similar individuals. They faced dilemmas about breastfeeding in public due to cultural and economic factors and found influential family members and broader community strategies might be more effective in promoting breastfeeding. Additionally, many women felt they lacked the professional support they needed.
McFadden et al. (2013)	High	Peer support; community	Breastfeeding/chest feeding (not specified)	Race/ethnicity/culture; Language; Religion	Interview; Focus group	**Reach and Recruitment:** Some women skipped breastfeeding support groups due to time constraints, discomfort with public breastfeeding, or lack of need, revealing gaps in culturally sensitive support. **Interaction:** While women were satisfied with early post‐natal breastfeeding support from health professionals, some felt visits were too brief and rushed, leading to a perception of a “tick box” approach rather than personalised care.
Mengoni et al. (2023)	High	Peer support	Breastfeeding/chest feeding (not specified)	Disability	Interview	**Reach and recruitment:** Accessing specialist support for children with specific needs, such as disabilities, posed additional challenges, particularly when these needs fell outside the scope of existing services. **Interaction:** Mothers valued peer and professional support for meeting breastfeeding goals but faced challenges with inconsistent professional knowledge and support for children with Down's syndrome.
Newman and Williamson (2018)	High	Community	Breastfeeding/chest feeding (not specified)	Race/ethnicity/culture; Longer term breastfeeding	Interview	**Interaction:** Breastfeeding groups and online platforms boosted women's confidence, provided support, and facilitated connections with other mothers, though specialist groups were preferred to avoid stigma and negative feedback.
Psarros (2018)	High	Peer support; community	Breastfeeding/chest feeding (not specified)	Race/ethnicity/culture; age	Focus group	**Interaction:** Several women successfully breastfed after community support referrals, highlighting the importance of trusted VCSEs and peer support networks, especially when initial hospital support was insufficient. **Overall experience:** Prejudice towards young mothers led to varying levels of support for breastfeeding, with some experiencing neglect or undermining from staff, partly due to stigma related to their age and mental health.
Raine (2003)	High	Peer support; community	Breastfeeding/chest feeding (not specified)	Socioeconomic status; Social capital	Interview; Participant observation; Diaries	**Interaction:** While the project may not drastically change breastfeeding rates immediately, it might foster valuable partnerships between health professionals and lay supporters, enhance support for mothers, and highlight the need for ongoing advocacy to integrate lay supporters in health initiatives.
Scott et al. (2003)	High	Peer support	Breastfeeding/chest feeding (not specified)	Socioeconomic status	Focus group	**Interaction:** Women valued the immediate, empathetic assistance and consistent support from peers who had firsthand experience, which significantly helped them overcome breastfeeding challenges.
Thompson et al. (2020)	High	Peer support; community	Breastfeeding/chest feeding (not specified)	Longer term breastfeeding	Interview	**Interaction:** Women felt societal attitudes and lack of quality support negatively impacted breastfeeding, with peer groups offering crucial acceptance and practical advice often lacking from professionals.
Thomson et al. (2012, 2015)	Medium	Peer support	Breastfeeding/chest feeding initiation; Breastfeeding/chest feeding maintenance	Race/ethnicity/culture	Interview	**Reach and recruitment:** There were challenges in accessing certain groups, particularly some Asian communities. **Sustainment:** Ongoing perinatal support allowed women to seek relevant information on personal and physical breastfeeding barriers, with Star Buddies' practical experience and shared understanding enhancing trust and effective support. **Interaction:** The role of peer supporters in building social capital across diverse backgrounds, fostering trust through shared experiences, and offering practical, culturally aware, and ongoing support to address individual breastfeeding challenges and goals was helpful.
Thomson et al. (2012)	High	Peer support	Breastfeeding/chest feeding initiation; Breastfeeding/chest feeding maintenance	Socioeconomic status	Interview; Focus group	**Interaction:** Incentives like gift schemes helped build strong, regular connections between peer supporters and women, facilitating ongoing support and reducing feelings of isolation for the women by ensuring frequent, meaningful interactions.
Thomson and Crossland (2013)	High	Peer support	Breastfeeding/chest feeding (not specified)	Mixed feeding	Interview	**Reach and Recruitment:** Most women reported successful initial contact with the helpline, but some experienced long wait times and multiple attempts, leading to frustration and stress. **Interaction:** Women felt empowered by the helpline's knowledge and support, which provided new insights, bolstered their confidence to challenge advice, and served as a reassuring lifeline, reducing feelings of isolation and confusion.
Trickey (2018)	High	Peer support	Breastfeeding/chest feeding (not specified)	Socioeconomic status	Interview; Focus group; Creative methods	**Interaction:** Peer supporters, valued for their nonprofessional, woman‐centred approach, enabled mothers to ask questions freely, but sustaining support groups in low‐income areas was challenging due to cultural norms and lack of breastfeeding prevalence, necessitating neutral venues to avoid exclusion.
Wagg et al. (2021, 2022)	High	Peer support; community	Breastfeeding/chest feeding (not specified)	Longer term breastfeeding	Interview	**Interaction:** Women valued online breastfeeding support groups for their accessibility, confidence‐building, and ability to provide both informational and experiential support, especially when face‐to‐face options felt intimidating or inadequate.

### 3.5 Reach and Recruitment

Seventeen studies, with 25 associated reports, explored reach and recruitment. Eight studies reported on peer support models, while six considered community provision, and three reported on both. Studies primarily discussed reach and recruitment in terms of intervention awareness and access.

Women and health professionals indicated general service unawareness, especially when services were newly introduced to the system (Beake et al. 2005; Brown et al. 2021; Brown and Tennant‐Eyles 2021; Carrington‐Windo 2018). In terms of specific population groups, there was limited knowledge about eligibility. Mothers who mixed‐fed misperceived that support only targeted exclusive breastfeeding (Thomson et al. 2022), or health visitors did not know if community approaches would meet the needs of Black and minority ethnic communities (Brown et al. 2021). Interventions adopted strategies to mitigate these issues by providing resource leaflets that extended women's knowledge (Ingram et al. 2020), or introducing support workers in socio‐economically deprived areas to proactively reach out and identify women's needs (Beake et al. 2005).

Experiences of access were mixed, with some studies reporting positive experiences (Clarke et al. 2020). However, there were barriers, including: a lack of service frequency (Fraser et al. 2020); limited provision during the nighttime or weekends (Brown et al. 2021; Thomson and Crossland 2013); inaccessible locations (Brown and Tennant‐Eyles 2021; Morse and Brown 2021); or inadequate geographical reach (Aiken and Thomson 2013).

Inequity in access was primarily reported in relation to language, culture and ethnicity, and socioeconomic deprivation. There was a consistent lack of cultural tailoring to migrated communities (Cook et al. 2021; Thomson et al. 2015). In one study of community breastfeeding support for women of Bangladeshi origin, authors noted that it was other family members, rather than the breastfeeding women, who needed to be initially approached:

For example, one community midwife said: I think there's the permission thing, as well. Often you have to go by custom, you know, mother‐in‐law for them to get permission to go to things

(practitioner focus group four) (McFadden et al. 2013)

Studies reported challenges to access for women from relatively deprived communities (McFadden et al. 2013; Islam 2016). There were individual‐level barriers, driven by socio‐cultural and economic factors: lack of confidence; childcare commitments; or having no private or reliable public transport (Brown and Tennant‐Eyles 2021; Hunt et al. 2021). Furthermore, interventions were thought to be based on middle class assumptions, such as women having partners who could take leave and support intervention uptake:

It can be incredibly hard to engage people in real deprivation areas […] to come out to face to face services even at a Children's Centre, you know it is, that is really quite challenging, and those with more resources in terms of confidence and wider experience maybe find it easier to access services

(Sophie, Org C) (Hunt et al. 2021)

There were additional considerations about accessing specialist support for children who had specific needs (e.g. disability) and did not fall within the remit of existing services (Mengoni et al. 2023). Where tailored support was needed, women had to advocate for provision, which could be difficult if they were an inexperienced breast feeder (Jackson and Hallam 2019).

### 3.6 Retention

Four studies, with 10 associated reports, included data related to retention and drop‐out. Three studies were focused on peer support and one targeted both community and peer provision.

Women reported geographical challenges to maintaining engagement, with the perceived inconvenience of travel to peer supporters potentially leading to a hesitancy to contact them:

It's meant to be like a service that you can call out and that you can have support, and if you think oh no I won't call her because she's going to need an hour on the bus then that's really not fair on her, it's not that bad and we'll wait

(Partner of P2—intervention, site A) (Clarke et al. 2020)

This was reflected by peer supporters themselves, who observed that women's homes could be a “nightmare” to reach and so “you spend half your time travelling” (Clarke et al. 2020). Continued engagement could also be an issue in rural areas due to ongoing inaccessibility (Brown and Tennant‐Eyles 2021; Morse and Brown 2021; Trickey 2018).

In areas of socioeconomic deprivation, peer support groups were often only sustained by middle‐class mothers journeying from outside of the local area, which created a cultural context that alienated lower‐income women and encouraged drop‐out (Trickey 2018). Equally, there were reported challenges in ensuring ongoing venue use for breastfeeding groups that could be reached by public transport and secured at low or no cost (Brown and Tennant‐Eyles 2021). This was a particular issue with the withdrawal of funding from community provisions such as Children's Centres (Jackson and Hallam 2019).

The Covid‐19 pandemic was observed to have an impact on retention as provision transitioned online (Brown and Tennant‐Eyles 2021). Peer supporters noted that attendance declined as mothers experienced online fatigue. Lack of continued support was potentially more detrimental to women with lower educational attainment, from Black and minority ethnic backgrounds, and experiencing more challenging living arrangements, as they were more likely to report lockdown had adversely impacted their capacity to breastfeed.

### 3.7 Interaction

Fifty‐four studies, with 67 study reports, included data on intervention interaction. Nineteen studies reported on community support, 22 studies focused on peer support, and 13 studies included both peer and community provision Studies primarily focused on women's challenging experiences during intervention participation.

Socioeconomic deprivation was the most explored characteristic. Within lower‐income areas, formula feeding was consistently seen as culturally normative, and considered important in facilitating a shared community of care with other family members (Trickey 2018). As such, there could be a lack of widespread encouragement to breastfeed. Within this context, women could feel unable or unconfident to ask for help in interacting with services (Hoddinott et al. 2010; Brown et al. 2021, #62). Having a peer supporter accompany them to a breastfeeding group could be helpful (Clarke et al. 2020; Thomson et al. 2012). Moreover, this peer support was often seen as a vital substitute for a naturally occurring support system and could remove feelings of isolation (Raine 2003).

Individuals from diverse ethnic groups reported numerous challenges to intervention interaction, often related to entrenched beliefs and relationship networks. For example, cultural taboos within the Irish Traveller community about being seen breastfeeding made it difficult to persist when living in accommodation where people were often coming in and out (Condon and Salmon 2015). Meanwhile, Bangladeshi women explained that other women within their families had influence over their feeding decisions, which could potentially negate intervention messaging (McFadden and Toole 2006).

Despite common experiences across various ethnic groups, relying on cultural stereotypes was acknowledged as preventing women's support needs being met (McFadden et al. 2013). While many voluntary organisations were working closely with different communities to understand their specific needs (Psarros 2018), peer supporters were sometimes unprepared for dealing with cultural differences (Brown and Tennant‐Eyles 2021). A common theme was that women felt peer and community support from those who shared their social, cultural, language and age characteristics could prevent misunderstandings in experiences and needs (Beake et al. 2005).

Additional underserved groups, while less frequently addressed, reported a range of needs that were not well understood or catered for by intervention. Women who sought support for breastfeeding babies with Down's Syndrome found a lack of system knowledge about how to overcome feeding barriers (Cartwright and Boath 2018; Mengoni et al. 2023). For nonbinary people who had given birth, there was a paucity of accessible, specialised support, particularly for pain and feeding alongside body dysphoria and hormone therapy (Jackson et al. 2024). Furthermore, women with a higher Body Mass Index (BMI) (≥ 30 kg/m^2^) faced difficulties with the lack of tailored information, with suggested breastfeeding positions being problematic with larger breasts and body size (Lyons et al. 2019). Age was also cited as a source of judgement for some younger mothers (Cook et al. 2021), where an expectation that they would not breastfeed potentially leading to lower levels of support (Psarros 2018).

Studies also considered inequity generating experiences in specific relation to the breastfeeding journey. For mothers engaging in breastfeeding beyond early infancy, there was a perception that healthcare professionals were misinformed or lacking knowledge about longer‐term breastfeeding (Jackson and Hallam 2019; Thompson et al. 2020). In such instances, women often felt online communities provided relevant expertise (Jackson and Hallam 2021), with the most appropriate support coming from mothers in a similar situation.

Mothers who decided to use mixed‐feeding sometimes reported experiencing negative reactions from breastfeeding supporters (Thomson and Crossland 2013). In many cases, this experience clearly intersected with women's socioeconomic status. Where more socio‐economically deprived women elected to formula feed or use mixed‐feeding following breastfeeding, they could feel reluctant to attend baby groups in more affluent areas characterised by high rates of breastfeeding, because they felt they would be judged (Gallagher 2023).

Moving beyond the perspectives of specific population groups, studies also considered how intervention experiences and needs could differ in relation to frequency and timing of interactions, often highlighting the need for personalised tailoring. Women varied in the level of proactive support they felt comfortable with. For example, there were cases of individuals finding frequent “checking in” phone calls helpful (Battersby 2001), while others did not appreciate such a volume of contact:

One of the other mums said it was too much… whereas another mum loved it, and just lapped it up, she could have been visited 100 times and would have enjoyed it.

(Health Professional 001) (Paranjothy et al. 2017)

From the peer supporters' perspective, greater frequency was associated with more meaningful relationships that enabled them to discuss issues around breastfeeding as well as other social and health difficulties (Thomson et al. 2012).

### 3.8 Sustainment

Seven studies, linked to 12 study reports, considered sustainment. Five studies focused on peer support, one on community provision and one on both community and peer support. Sustainment was discussed in terms of how intervention mechanisms and outcomes could be maintained post‐intervention.

Studies often emphasised the importance of reconfiguring and strengthening social networks, as they were seen as vital in providing enduring encouragement for breastfeeding continuity:

…mums then become part of a, communal supportive network, that has all sorts of other related benefits…and you know helping them to continue to feed for longer if that's what they're choosing to do.

(Yvonne, Org C) (Hunt 2019)

These networks could be generated within specific interventions, such as community groups, or through participants' wider communities. Peer supporters were seen as particularly productive in building sustained social capital around breastfeeding practices. In one study it was observed that peer supporters' own success in overcoming issues and determining to help others could set off a ‘ripple’ effect of women offering and receiving support within their social circle (Hunt 2019). However, the study noted that while support could augment breastfeeding, ultimately without the restructuring of cultural norms within home and communities (e.g. pro‐bottle‐feeding attitudes), eventually pre‐existing barriers would re‐emerge.

These barriers also evolved over time, and returning to work was a critical point that could compromise breastfeeding sustainment (Jackson and Hallam 2021). Practical, pragmatic advice such as on expressing milk (Wade et al. 2009) and a supportive work environment (Hunt 2019) were important for supporting women to continue breastfeeding*:*

I was planning on 6 months [breastfeeding] and then going back to work… [But] the [Star Buddies] have given me the confidence to know that I can feed her in the morning and at night that my body will regulate and I can then still feed as normal at the weekends.

(Mary) (Thomson et al. 2012)

Importantly, it was recognised that positive intervention effects may not be fully observed unless women have additional babies. For example, in areas with a strong culture of bottle‐feeding, a positive experience of breastfeeding support services, regardless of current feeding decision, may increase future intention to breastfeed (Brown et al. 2021). As such, the timing of intervention outcomes, and the activation of their mechanisms, may occur years beyond participation experience.

### 3.9 Confidence in Evidence

We generated eight evidence statements that integrated evidence from across the four domains and the cross‐cutting principles presented in the discussion (Table 6). We assessed six statements as having high confidence in findings and two as having moderate confidence.

**Table 6:** CERQual summary of findings.

Summarised review finding	GRADE‐CERQual assessment of confidence	Explanation of GRADE‐CERQual assessment	References
There is a lack of awareness of peer and community support, with limited knowledge of new provisions or eligibility criteria among professionals.	High confidence	**Very minor concerns regarding methodological limitations:** The quality of five of six studies was high. Methodological challenges did not significantly affect the quality of the evidence supporting the finding. Three studies did not incorporate a theoretical perspective. In one study, the setting and data collection method weren't adequately described. **Very minor concerns regarding coherence:** Studies presented consistent and complementary results. **Very minor concerns regarding adequacy:** There was an adequate number of studies supporting this statement across different populations in the UK. **Very minor concerns regarding relevance:** The results explicitly address awareness among underserved populations.	Beake et al. (2005), Brown et al. (2021), Brown and Tennant‐Eyles (2021), Carrington‐Windo (2018), Ingram et al. (2020), Thomson et al. (2022).
Access to intervention is inhibited by lack of frequency, limited hours of availability, inaccessible locations, and inadequate geographical reach. Women from lower socioeconomic backgrounds can struggle to maintain access where they are reliant on public transport or funding from low‐cost community spaces is withdrawn.	High confidence	**No/Very minor concerns regarding methodological limitations:** Eleven of the 13 studies were high quality. Methodological challenges did not significantly affect the quality of the evidence supporting the finding. Studies did have a limited theoretical perspective. **No/Very minor concerns regarding coherence:** Studies had a largely coherent narrative regarding access and recruitment. There was one negative case, where women reported negative recruitment experiences into the intervention. Experiences of access were both positive and negative within and across studies, but descriptions and explanations were largely coherent. **No/Very minor concerns regarding adequacy:** All studies discussed reach, recruitment and access in detail, providing rich description and data. **No/Very minor concerns regarding relevance:** Studies discussed how different populations experienced challenges in accessing intervention.	Aiken and Thomson (2013), Brown et al. (2021), Brown and Tennant‐Eyles (2021), Clarke et al. (2020), Thomson and Crossland (2013), Cook et al. (2021), Fraser et al. (2024), Islam (2016), Jackson and Hallam (2019), Hunt et al. (2021), Hunt (2019), McFadden et al. (2013), Mengoni et al. (2023), Morse and Brown (2021), Thomson et al. (2015, 2020)
Community groups can feel tailored to middle‐class values, which can be particularly alienating to women from more socio‐economically deprived areas. It can be particularly challenging when women elect to bottle or mix‐feed.	Moderate confidence	**Minor concerns regarding methodological limitations:** Two out of three studies had high methodological quality. Methodological challenges did not significantly affect the quality of the evidence supporting the finding. One of the three studies raised some issues regarding generalisability. **No/Very minor concerns regarding coherence:** Studies were consistent and coherent in reporting on the alienation of women. There were no clear negative cases. **Minor concerns regarding adequacy since:** Studies provided sufficient data, although were limited in number. **No/Very minor concerns regarding relevance:** Studies reported data from underserved populations who were primarily from lower socioeconomic backgrounds.	Fox et al. (2015), Gallagher (2023), Hunt et al. (2021), Thompson et al. (2020), Thomson et al. (2015), Trickey (2018).
Individuals from diverse ethnic groups, in addition to those with different physical characteristics (e.g. larger bodies) or at varying points in their breastfeeding journey (e.g. longer‐term feeding), feel their specific needs are often not met, and professionals often do not have the requisite knowledge and skill.	High confidence	**Minor concerns regarding methodological limitations:** Three of the 12 studies did not describe the setting or social characteristics and had limited generalisability. A theoretical perspective was not explicit in six studies. However, this did not significantly affect the quality of the evidence supporting the finding. **No/Very minor concerns regarding coherence**: Studies were consistent and coherent in their findings. **No/Very minor concerns regarding adequacy:** All studies that supported this finding provided adequate data to describe and explain the experiences of different populations. **No/Very minor concerns regarding relevance:** Studies reported data from underserved populations.	Beake et al. (2005), Cartwright and Boath (2018), Cook et al. (2021), Gallagher (2023), Jackson and Hallam (2021), Jackson et al. (2024), Lyons et al. (2019), Psarros (2018), McFadden et al. (2013), Mengoni et al. (2023), Thompson et al. (2020), Thomson and Crossland (2013).
Strong social networks can encourage women to sustain breastfeeding, with peer supporters having a key role in catalysing systems of support within these networks.	Moderate confidence	**No/Very minor concerns regarding methodological limitations.** The quality of this study was high. **Moderate concerns regarding coherence**: There is only contributing study so coherence across evidence sources cannot be assessed. **Moderate concerns regarding adequacy:** Though the finding and its context are clear, there are moderate concerns as there is only one contributing study. **No/Very minor concerns regarding relevance:** Although there is only one contributing study, the data are relevant.	Hunt (2019), Hunt et al. (2021).
To support the maintenance of intervention mechanisms and outcomes, there is a need for wider restructuring of the system so that cultural norms and resources within families and communities support breastfeeding.	High confidence	**No/Very minor concerns regarding methodological limitations**: Methodological challenges did not significantly affect the quality of the evidence supporting the finding. **No/Very minor concerns regarding coherence:** Studies were coherent and consistent in their findings. **No/Very minor concerns regarding adequacy:** All studies provided rich description and explanation of their findings. **No/Very minor concerns regarding relevance:** Studies reported data from underserved populations.	Brown et al. (2021), Jackson and Hallam (2019), Hunt (2019), Thomson et al. (2012), Wade et al. (2009).
When viable, it is preferable for peer supporters to share social characteristics with the individuals they are supporting.	High confidence	**No/Very minor concerns regarding methodological limitations**: Methodological challenges did not significantly affect the quality of the evidence supporting the finding. Three of the 15 studies were not clear about the generalisability of their findings. **No/Very minor concerns regarding coherence:** Studies were consistent and coherent in their findings. There was some complexity as mothers had different needs around the frequency and nature of the supporting relationship. And stakeholders identified it could be practically difficult to achieve. **Minor concerns regarding adequacy:** The required identity of peer supporters was explicitly discussed in rich detail in twelve of the studies. **No/Very minor concerns regarding relevance:** Studies reported data from underserved populations.	Beake et al. (2005), Cartwright and Boath (2018), Cook et al. (2021), Gallagher (2023), Hunt et al. (2021), Jackson and Hallam (2021), Jackson et al. (2024), Lyons et al. (2019), McFadden et al. (2013), McFadden and Toole (2006), Mengoni et al. (2023), Psarros (2018), Thompson et al. (2020), Thomson et al. (2015), Thomson and Crossland (2013).
Interventions need to be tailored to the needs of individuals. It is important to avoid drawing on stereotypes and understand the needs of particular population groups.	High confidence	**No/Very minor concerns regarding methodological limitations.** Methodological challenges did not significantly affect the quality of the evidence supporting the finding. Seven of the studies did not have a clear theoretical perspective. **No/Very minor concerns regarding coherence:** Studies were coherent and consistent in their findings. **No/Very minor concerns regarding adequacy:** All five studies provided rich description and explanation of their findings. **No/Very minor concerns regarding relevance:** Studies reported data from underserved populations.	Battersby (2001), Beake et al. (2005), Brown et al. (2021), Brown and Tennant‐Eyles (2021), Cartwright and Boath (2018), Cook et al. (2021), Fox et al. (2015), Hoddinott et al. (2010), Jackson et al. (2024), Lyons et al. (2019), McFadden et al. (2013), Mengoni et al. (2023), Psarros (2018), Raine (2003), Regan and Brown (2019), Thompson et al. (2020), Thomson et al. (2012), Trickey (2018).

## 4 Discussion

This systematic review synthesised the existing evidence‐base reporting participants' experiences of peer and community breastfeeding interventions in the UK, to identify the potential for inequities. Notably, we disaggregated the overall experience, moving beyond the generic concept of “acceptability” to explore reach and recruitment, retention, interaction and sustainment. This approach adds nuance and could be used as a structuring framework for future qualitative research, evaluation and synthesis (Moore et al. 2015; Skivington et al. 2021).

We identified challenges across intervention phases, which may contribute to negative experiences and cause inequities. While impacting various underserved populations, these challenges were most frequent for women from lower socioeconomic backgrounds and Black and minority ethnic communities. There was limited awareness of interventions and their eligibility criteria. Lack of provision availability by time, day, or suitable locations could inhibit uptake. Interventions tended to be inadequately tailored to individual needs, with professionals often not being sure how to achieve cultural sensitivity. Finally, there were wider structural barriers to the maintenance of breastfeeding practices, with a failure to provide supportive social networks leading to the re‐emergence of non‐breastfeeding promoting cultural norms once interventions tapered off.

### 4.1 Principles for Future Peer and Community Intervention

**Figure 3:**
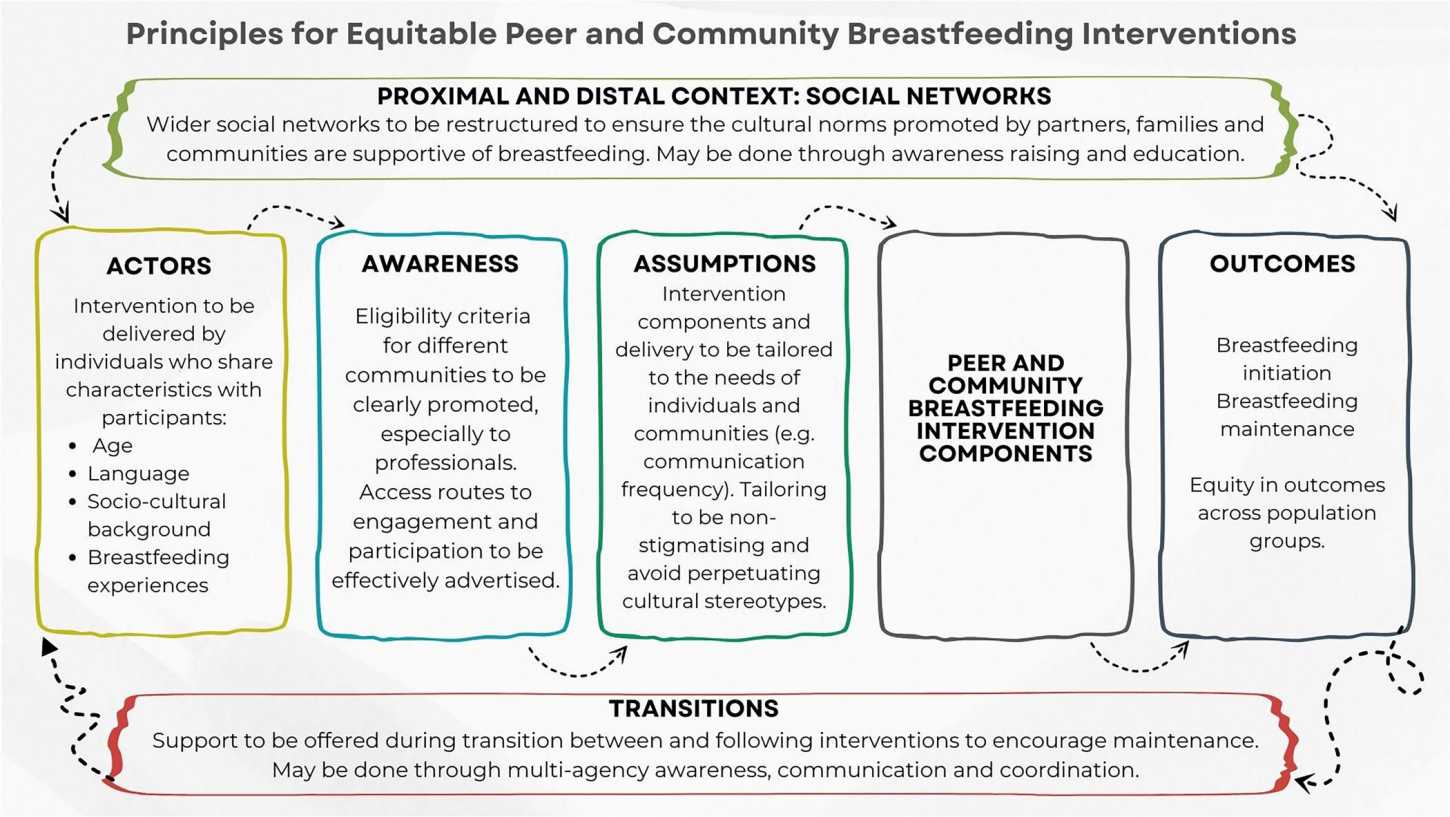
Principles for equitable peer and community breastfeeding interventions.

Drawing on the studies included in the review, combined with discussion from stakeholder consultation, we identified five principles to support future breastfeeding interventions in being equitable. We have combined these in an overarching model (Figure 3), offering a set of guiding principles that could be applied to a range of peer and community breastfeeding activities. These principles draw upon and reinforce recommendations endorsed in guidance issued by prominent international organisations such as the World Health Organisation (World Health Organisation 2018) and UNICEF (UNICEF 2023).

#### 4.1.1 Actors

Community and peer support should ideally be delivered by individuals of a similar age, language or cultural background. This can also extend to being supported by women who have also breastfed (Beake et al. 2005). Relationships with peer supporters might also be strengthened if they are in spaces (e.g. centres for homeless) that women already frequent. However, it is important to acknowledge that recruiting and retaining appropriate peer supporters is challenging, and in socio‐economically deprived communities there have been issues with complex social needs and multiple competing pressures (Trickey 2018). Within a contemporary context of “cost of living” crises, it is further important to recognise that some individuals may choose or need to pursue paid rather than voluntary opportunities.

#### 4.1.2 Awareness

The eligibility criteria and access routes for interventions need to be effectively promoted, particularly among professionals. Baby Friending Initiatives Standards for Health Services mandates an educated workforce (UNICEF), and knowledge of local and national community and peer provision needs to be part of this. Further work may be required to identify effective routes for promoting awareness across the breastfeeding journey, potentially with a view to reducing reliance on leaflets and posters.

#### 4.1.3 Assumptions

Intervention messaging and delivery should assume that tailoring will be required to meet individual and community needs (Hoddinott et al. 2010). This tailoring should be non‐stigmatising to avoid perpetuating cultural stereotypes (McFadden et al. 2013). However, while different delivery modalities and providers can ensure timely support, there are risks of some community forums providing misinformation (Fox et al. 2015; Regan and Brown 2019).

#### 4.1.4 Transitions

In practice, interventions do not operate in isolation and women may transition between provision. For example, women may be more inclined to move to increased levels of support (e.g. peer support) if they have a positive connection with less intense provision (e.g. helplines) (Fox et al. 2015; Hunt et al. 2021) or behavioural maintenance may be improved where women transition from one‐to‐one support into group‐based approaches (Copeland et al. 2019; Ingram 2013). Successful transitions can avoid the risk of abrupt withdrawals of support, which can be disorientating or discouraging. To this end, it is imperative to have effective multi‐agency awareness, communication and coordination to ensure women can successfully make these transitions.

#### 4.1.5 Social Networks

The wider system needs to be restructured, with support to ensure the cultural norms promoted by partners, families and communities are encouraging of breastfeeding. This may be done through awareness raising and education across the course of pregnancy and breastfeeding, with research highlighting the importance and value of including partners and significant others in antenatal education (Ingram et al. 2003; Ogbo et al. 2016; Rempel and Rempel 2011). Services themselves need to understand how existing social capital can be built upon (Thomson et al. 2015).

### 4.2 Future Research

There are identified evidence gaps that need to be addressed in future. First, there is an emergent qualitative evidence‐base for some key underserved social characteristics identified as important to stakeholders, notably neurodiversity, that are not yet being captured by intervention research in the UK (Grant et al. 2022). Second, the majority of interventions were focused on breastfeeding, with limited consideration of other feeding approaches. Stakeholders felt that work is required to understand how women and birthing people may be supported to safely bottle‐feed and mix‐feed in a non‐stigmatising manner, and the potential for inequities that might arise when seeking supporting these approaches. Third, while exploring participants' experiences is imperative to understanding how inequities might emerge, there remains a need to conduct component analysis to elicit which activities contribute to these inequities.

### 4.3 Review Limitations

There are limitations that should be considered when interpreting the review. First, we were provided with a funding remit to synthesise evidence on peer support and community interventions. Stakeholder consultation indicated it was challenging to define these intervention types. We observed this when assessing eligibility, with the difficulty compounded by a lack of systematic description of interventions in accordance with the Template for Intervention Description and Replication (TIDieR) checklist (Hoffmann et al. 2014). As such we may not have identified some interventions or those identified could have been classified differently. Second, it could be challenging to define studies as high or low equity, as it was difficult to decide if studies were explicitly focused on underserved populations or if the study sample happened to represent a particular group (e.g. lower socioeconomic communities). This may present some challenges for the future replicability and updating of the review.

## 5 Conclusion

This systematic review of peer and community breastfeeding interventions offers one of the first syntheses of inequities in participants' experiences. In differentiating four phases of intervention experience, the review demonstrates issues across the life course of participation and outcome maintenance. Future intervention development and delivery needs to be sensitised to the social characteristics and needs of different population groups.

## Author Contributions

R.E., S.R., J.C., S.H., J.S.C., R.G., J.T.C., K.J., K.M., and G.M.T. conceived the review. J.K. and S.R. conducted all information specialist related activity. R.E., C.D., R.A., J.K., S.R., J.C., S.H., S.L., J.S.C., and G.M.T. undertook study screening data extraction, and quality appraisal. J.C., S.H., J.S.C., K.J., and K.M. supported recruitment for stakeholder engagement. R.E. and R.A. led the stakeholder engagement sessions. R.E., C.D., R.A., and J.K. drafted the synthesis. All member of team confirmed the synthesis. R.E. and C.D. drafted the manuscript. Figures, tables and supplements were prepared by RE, C.D., R.A., J.K., and S.R. All members of the team confirmed the manuscript.

## Disclosure

Prof G.J. Melendez‐Torres is an NIHR Senior Investigator. K.J. and J.C. have undertaken research on peer support for breastfeeding and are investigators on the ongoing ABA‐feed randomised controlled trial (NIHR129182).

## Supporting information

ENTREQ Reporting Guidelines.

Supplement A.

Supplement B.

Supplement C.

## Data Availability

The authors have nothing to report.
