# Supplementary material for: Peer Support and Community Interventions Targeting Breastfeeding in the UK: Systematic Review of Qualitative Evidence to Identify Inequities in Participants' Experiences
Source: Matern Child Nutr. 2025 May 19;21(4):e70041. doi: 10.1111/mcn.70041 (PMC12454195; doi:10.1111/mcn.70041)
Supplement: Supplementary file 2 — Supplement A. [file MCN-21-e70041-s004.docx]

**Supplement A**

*Database: Epistemonikos (For identification of systematic reviews)*

| **#** | **Query** | **Results from 16 Jan 2024** |
| --- | --- | --- |
| 1 | (title:(breastfe*) OR abstract:(breastfe*)) OR (title:(breast-fe*) OR abstract:(breast-fe*)) OR (title:((breast AND feed*)) OR abstract:((breast AND feed*))) OR (title:((breast AND fed)) OR abstract:((breast AND fed))) |  |
| 2 | Publication year: Last 5 years |  |
| 3 | Publication type: Systematic review | 1101 |

*Overview of bibliographic database searches*

| **Date** | **Database name** | **Number of hits** |
| --- | --- | --- |
| 19/03/24 | Medline (Ovid) | 650 |
| 20/03/24 | Embase (Ovid) | 1,201 |
| 18/03/24 | PsycInfo (Ovid) | 159 |
| 19/03/24 | CINAHL (Ebsco) | 142 |
| 18/03/24 | BNI (ProQuest) | 87 |
| 19/03/24 | Scopus | 225 |
| 18/03/24 | ASSIA (ProQuest) | 104 |
| 20/03/24 | Social Policy and Practice (Ovid) | 42 |
|  |  |  |
|  | **Total** | 2,610 |
|  | **Duplicates** | 778 |
|  | **Total to screen** | 1,832 |

*Database: Ovid MEDLINE(R) ALL <1946 to March 15, 2024>*

| **#** | **Query** | **Results from 18 Mar 2024** |
| --- | --- | --- |
| 1 | exp Breast Feeding/ | 44,968 |
| 2 | exp Lactation/ | 49,040 |
| 3 | (breastfeed* or breast feed* or breastfed* or breast fed or breastmilk or breast milk or expressed milk* or chestfeed* or chest feed* or bodyfeed* body feed* or chest fed or body fed).ti,ab,kf. | 67,100 |
| 4 | (nursing adj2 (baby or infant* or newborn* or mother* or parent* or birthing people or birthing person*)).ti,kf. | 960 |
| 5 | ((infant* or baby or babies or newborn*) adj3 (milk or fed or feed* or lactat*)).ti,ab,kf. | 26,375 |
| 6 | lactation.ti,kf. | 17,749 |
| 7 | 1 or 2 or 3 or 4 or 5 or 6 | 133,567 |
| 8 | (interview: or experience:).mp. or qualitative.tw. | 1,972,746 |
| 9 | ((("semi-structured" or semistructured or unstructured or informal or "in-depth" or indepth or "face-to-face" or structured or guide) adj3 (discussion* or questionnaire*)) or (focus group* or ethnograph* or fieldwork or "field work" or "key informant")).ti,ab. or interviews as topic/ or focus groups/ or narration/ or qualitative research/ | 253,859 |
| 10 | 8 or 9 | 2,027,297 |
| 11 | exp United Kingdom/ | 393,442 |
| 12 | (national health service* or nhs*).ti,ab,in. | 286,666 |
| 13 | (english not ((published or publication* or translat* or written or language* or speak* or literature or citation*) adj5 english)).ti,ab. | 125,236 |
| 14 | (gb or "g.b." or britain* or (british* not "british columbia") or uk or "u.k." or united kingdom* or (england* not "new england") or northern ireland* or northern irish* or scotland* or scottish* or ((wales or "south wales") not "new south wales") or welsh*).ti,ab,jw,in. | 2,529,167 |
| 15 | (bath or "bath's" or ((birmingham not alabama*) or ("birmingham's" not alabama*) or bradford or "bradford's" or brighton or "brighton's" or bristol or "bristol's" or carlisle* or "carlisle's" or (cambridge not (massachusetts* or boston* or harvard*)) or ("cambridge's" not (massachusetts* or boston* or harvard*)) or (canterbury not zealand*) or ("canterbury's" not zealand*) or chelmsford or "chelmsford's" or chester or "chester's" or chichester or "chichester's" or coventry or "coventry's" or derby or "derby's" or (durham not (carolina* or nc)) or ("durham's" not (carolina* or nc)) or ely or "ely's" or exeter or "exeter's" or gloucester or "gloucester's" or hereford or "hereford's" or hull or "hull's" or lancaster or "lancaster's" or leeds* or leicester or "leicester's" or (lincoln not nebraska*) or ("lincoln's" not nebraska*) or (liverpool not (new south wales* or nsw)) or ("liverpool's" not (new south wales* or nsw)) or ((london not (ontario* or ont or toronto*)) or ("london's" not (ontario* or ont or toronto*)) or manchester or "manchester's" or (newcastle not (new south wales* or nsw)) or ("newcastle's" not (new south wales* or nsw)) or norwich or "norwich's" or nottingham or "nottingham's" or oxford or "oxford's" or peterborough or "peterborough's" or plymouth or "plymouth's" or portsmouth or "portsmouth's" or preston or "preston's" or ripon or "ripon's" or salford or "salford's" or salisbury or "salisbury's" or sheffield or "sheffield's" or southampton or "southampton's" or st albans or stoke or "stoke's" or sunderland or "sunderland's" or truro or "truro's" or wakefield or "wakefield's" or wells or westminster or "westminster's" or winchester or "winchester's" or wolverhampton or "wolverhampton's" or (worcester not (massachusetts* or boston* or harvard*)) or ("worcester's" not (massachusetts* or boston* or harvard*)) or (york not ("new york*" or ny or ontario* or ont or toronto*)) or ("york's" not ("new york*" or ny or ontario* or ont or toronto*))))).ti,ab,in. | 1,818,142 |
| 16 | (bangor or "bangor's" or cardiff or "cardiff's" or newport or "newport's" or st asaph or "st asaph's" or st davids or swansea or "swansea's").ti,ab,in. | 73,476 |
| 17 | (aberdeen or "aberdeen's" or dundee or "dundee's" or edinburgh or "edinburgh's" or glasgow or "glasgow's" or inverness or (perth not australia*) or ("perth's" not australia*) or stirling or "stirling's").ti,ab,in. | 267,541 |
| 18 | (armagh or "armagh's" or belfast or "belfast's" or lisburn or "lisburn's" or londonderry or "londonderry's" or derry or "derry's" or newry or "newry's").ti,ab,in. | 35,481 |
| 19 | or/11-18 | 3,240,034 |
| 20 | (exp africa/ or exp americas/ or exp antarctic regions/ or exp arctic regions/ or exp asia/ or exp australia/ or exp oceania/) not (exp United Kingdom/ or europe/) | 3,397,501 |
| 21 | 19 not 20 | 3,039,272 |
| 22 | 7 and 10 and 21 | 1,532 |
| 23 | limit 22 to (ed=20171201-20240401 or dt=20171201-20240401) | 650 |

*Database: Embase <1974 to 2024 March 15>*

| **#** | **Query** | **Results from 18 Mar 2024** |
| --- | --- | --- |
| 1 | exp breast feeding/ | 68,667 |
| 2 | exp *lactation/ | 23,583 |
| 3 | (breastfeed* or breast feed* or breastfed* or breast fed or breastmilk or breast milk or expressed milk* or chestfeed* or chest feed* or bodyfeed* body feed* or chest fed or body fed).ti,ab,kf. | 83,047 |
| 4 | (nursing adj2 (baby or infant* or newborn* or mother* or parent* or birthing person* or birthing people)).ti,kf. | 854 |
| 5 | ((infant* or baby or babies or newborn*) adj3 (milk or fed or feed* or lactat*)).ti,ab,kf. | 31,170 |
| 6 | 1 or 2 or 3 or 4 or 5 | 132,573 |
| 7 | interview:.tw. or exp health care organization/ or experiences.tw. | 2,900,013 |
| 8 | ((("semi-structured" or semistructured or unstructured or informal or "in-depth" or indepth or "face-to-face" or structured or guide) adj3 (discussion* or questionnaire*)) or (focus group* or ethnograph* or fieldwork or "field work" or "key informant")).ti,ab. or qualitative research/ | 257,117 |
| 9 | 7 or 8 | 2,986,226 |
| 10 | exp United Kingdom/ | 472,683 |
| 11 | (national health service* or nhs*).ti,ab,in,ad. | 490,049 |
| 12 | (english not ((published or publication* or translat* or written or language* or speak* or literature or citation*) adj5 english)).ti,ab. | 63,436 |
| 13 | (gb or "g.b." or britain* or (british* not "british columbia") or uk or "u.k." or united kingdom* or (england* not "new england") or northern ireland* or northern irish* or scotland* or scottish* or ((wales or "south wales") not "new south wales") or welsh*).ti,ab,jx,in,ad. | 3,812,687 |
| 14 | (bath or "bath's" or ((birmingham not alabama*) or ("birmingham's" not alabama*) or bradford or "bradford's" or brighton or "brighton's" or bristol or "bristol's" or carlisle* or "carlisle's" or (cambridge not (massachusetts* or boston* or harvard*)) or ("cambridge's" not (massachusetts* or boston* or harvard*)) or (canterbury not zealand*) or ("canterbury's" not zealand*) or chelmsford or "chelmsford's" or chester or "chester's" or chichester or "chichester's" or coventry or "coventry's" or derby or "derby's" or (durham not (carolina* or nc)) or ("durham's" not (carolina* or nc)) or ely or "ely's" or exeter or "exeter's" or gloucester or "gloucester's" or hereford or "hereford's" or hull or "hull's" or lancaster or "lancaster's" or leeds* or leicester or "leicester's" or (lincoln not nebraska*) or ("lincoln's" not nebraska*) or (liverpool not (new south wales* or nsw)) or ("liverpool's" not (new south wales* or nsw)) or ((london not (ontario* or ont or toronto*)) or ("london's" not (ontario* or ont or toronto*)) or manchester or "manchester's" or (newcastle not (new south wales* or nsw)) or ("newcastle's" not (new south wales* or nsw)) or norwich or "norwich's" or nottingham or "nottingham's" or oxford or "oxford's" or peterborough or "peterborough's" or plymouth or "plymouth's" or portsmouth or "portsmouth's" or preston or "preston's" or ripon or "ripon's" or salford or "salford's" or salisbury or "salisbury's" or sheffield or "sheffield's" or southampton or "southampton's" or st albans or stoke or "stoke's" or sunderland or "sunderland's" or truro or "truro's" or wakefield or "wakefield's" or wells or westminster or "westminster's" or winchester or "winchester's" or wolverhampton or "wolverhampton's" or (worcester not (massachusetts* or boston* or harvard*)) or ("worcester's" not (massachusetts* or boston* or harvard*)) or (york not ("new york*" or ny or ontario* or ont or toronto*)) or ("york's" not ("new york*" or ny or ontario* or ont or toronto*))))).ti,ab,in,ad. | 2,988,260 |
| 15 | (bangor or "bangor's" or cardiff or "cardiff's" or newport or "newport's" or st asaph or "st asaph's" or st davids or swansea or "swansea's").ti,ab,in,ad. | 123,033 |
| 16 | (aberdeen or "aberdeen's" or dundee or "dundee's" or edinburgh or "edinburgh's" or glasgow or "glasgow's" or inverness or (perth not australia*) or ("perth's" not australia*) or stirling or "stirling's").ti,ab,in,ad. | 411,189 |
| 17 | (armagh or "armagh's" or belfast or "belfast's" or lisburn or "lisburn's" or londonderry or "londonderry's" or derry or "derry's" or newry or "newry's").ti,ab,in,ad. | 57,421 |
| 18 | or/10-17 | 4,663,005 |
| 19 | (exp "arctic and antarctic"/ or exp oceanic regions/ or exp western hemisphere/ or exp africa/ or exp asia/ or exp "australia and new zealand"/) not (exp united kingdom/ or europe/) | 3,842,448 |
| 20 | 18 not 19 | 4,371,274 |
| 21 | 6 and 9 and 20 | 2,566 |
| 22 | limit 21 to (dd=20171201-20240401 or rd=20171201-20240401 or dc=20171201-20240401) | 1,201 |

*Database: APA PsycInfo <1806 to March Week 3 2024>*

| **#** | **Query** | **Results from 18 Mar 2024** |
| --- | --- | --- |
| 1 | breast feeding/ | 4,399 |
| 2 | lactation/ | 1,778 |
| 3 | (breastfeed* or breast feed* or breastfed* or breast fed or breastmilk or breast milk or expressed milk* or chestfeed* or chest feed* or bodyfeed* body feed* or chest fed or body fed).ti,ab,id. | 6,929 |
| 4 | (nursing adj2 (baby or infant* or newborn* or mother* or parent* or birthing people or birthing person*)).ti,id. | 154 |
| 5 | ((infant* or baby or babies or newborn*) adj3 (milk or fed or feed* or lactat*)).ti,ab,id. | 2,824 |
| 6 | lactation.ti,id. | 1,233 |
| 7 | 1 or 2 or 3 or 4 or 5 or 6 | 9,897 |
| 8 | experience:.mp. or interview:.tw. or qualitative:.tw. | 1,184,672 |
| 9 | ((("semi-structured" or semistructured or unstructured or informal or "in-depth" or indepth or "face-to-face" or structured or guide) adj3 (discussion* or questionnaire*)) or (focus group* or ethnograph* or fieldwork or "field work" or "key informant")).ti,ab. or qualitative research/ | 106,977 |
| 10 | 8 or 9 | 1,215,585 |
| 11 | "United Kingdom".mh. | 2,411 |
| 12 | United Kingdom.lo. | 107,665 |
| 13 | (national health service* or nhs*).ti,ab,in. | 30,701 |
| 14 | (english not ((published or publication* or translat* or written or language* or speak* or literature or citation*) adj5 english)).ti,ab. | 102,834 |
| 15 | (gb or "g.b." or britain* or (british* not "british columbia") or uk or "u.k." or united kingdom* or (england* not "new england") or northern ireland* or northern irish* or scotland* or scottish* or ((wales or "south wales") not "new south wales") or welsh*).ti,ab,jx,in. | 538,715 |
| 16 | (bath or "bath's" or ((birmingham not alabama*) or ("birmingham's" not alabama*) or bradford or "bradford's" or brighton or "brighton's" or bristol or "bristol's" or carlisle* or "carlisle's" or (cambridge not (massachusetts* or boston* or harvard*)) or ("cambridge's" not (massachusetts* or boston* or harvard*)) or (canterbury not zealand*) or ("canterbury's" not zealand*) or chelmsford or "chelmsford's" or chester or "chester's" or chichester or "chichester's" or coventry or "coventry's" or derby or "derby's" or (durham not (carolina* or nc)) or ("durham's" not (carolina* or nc)) or ely or "ely's" or exeter or "exeter's" or gloucester or "gloucester's" or hereford or "hereford's" or hull or "hull's" or lancaster or "lancaster's" or leeds* or leicester or "leicester's" or (lincoln not nebraska*) or ("lincoln's" not nebraska*) or (liverpool not (new south wales* or nsw)) or ("liverpool's" not (new south wales* or nsw)) or ((london not (ontario* or ont or toronto*)) or ("london's" not (ontario* or ont or toronto*)) or manchester or "manchester's" or (newcastle not (new south wales* or nsw)) or ("newcastle's" not (new south wales* or nsw)) or norwich or "norwich's" or nottingham or "nottingham's" or oxford or "oxford's" or peterborough or "peterborough's" or plymouth or "plymouth's" or portsmouth or "portsmouth's" or preston or "preston's" or ripon or "ripon's" or salford or "salford's" or salisbury or "salisbury's" or sheffield or "sheffield's" or southampton or "southampton's" or st albans or stoke or "stoke's" or sunderland or "sunderland's" or truro or "truro's" or wakefield or "wakefield's" or wells or westminster or "westminster's" or winchester or "winchester's" or wolverhampton or "wolverhampton's" or (worcester not (massachusetts* or boston* or harvard*)) or ("worcester's" not (massachusetts* or boston* or harvard*)) or (york not ("new york*" or ny or ontario* or ont or toronto*)) or ("york's" not ("new york*" or ny or ontario* or ont or toronto*))))).ti,ab,in. | 419,984 |
| 17 | (bangor or "bangor's" or cardiff or "cardiff's" or newport or "newport's" or st asaph or "st asaph's" or st davids or swansea or "swansea's").ti,ab,in. | 22,001 |
| 18 | (aberdeen or "aberdeen's" or dundee or "dundee's" or edinburgh or "edinburgh's" or glasgow or "glasgow's" or inverness or (perth not australia*) or ("perth's" not australia*) or stirling or "stirling's").ti,ab,in. | 51,917 |
| 19 | (armagh or "armagh's" or belfast or "belfast's" or lisburn or "lisburn's" or londonderry or "londonderry's" or derry or "derry's" or newry or "newry's").ti,ab,in. | 7,117 |
| 20 | 11 or 12 or 13 or 14 or 15 or 16 or 17 or 18 or 19 | 708,367 |
| 21 | 7 and 10 and 20 | 476 |
| 22 | limit 21 to up=20171201-20240401 | 159 |

*Database: CINAHL via EBSCOhost*

| **#** | **Query** | **Results from 18 Mar 2024** |
| --- | --- | --- |
| 1 | MW Breastfeeding OR MW lactation OR TI ( (breastfeed* or breast feed* or breastfed* or breast fed or breastmilk or breast milk or expressed milk* or chestfeed* or chest feed* or bodyfeed* body feed* or chest fed or body fed) ) OR AB ( (breastfeed* or breast feed* or breastfed* or breast fed or breastmilk or breast milk or expressed milk* or chestfeed* or chest feed* or bodyfeed* body feed* or chest fed or body fed) ) OR TI ( (nursing adj2 (baby or infant* or newborn* or mother* or parent* or birthing people or birthing person*) ) OR AB ( (nursing adj2 (baby or infant* or newborn* or mother* or parent* or birthing people or birthing person*) ) OR TI ( ((infant* or baby or babies or newborn*) adj3 (milk or fed or feed* or lactat*)) ) OR AB ( ((infant* or baby or babies or newborn*) adj3 (milk or fed or feed* or lactat*)) ) OR TI lactation ) |  |
| 2 | AND TX ( qualitative OR ethnol* OR ethnog* OR ethnonurs* OR emic OR etic OR leininger OR noblit OR "field note*" OR "field record*" OR fieldnote* OR "field stud*" or "participant observ*" OR "participant observation*" OR hermaneutic* OR phenomenolog* OR "lived experience*" OR heidegger* OR husserl* OR "merleau-pont*" OR colaizzi OR giorgi OR ricoeur OR spiegelberg OR "van kaam" OR "van manen" OR "grounded theory" OR "constant compar*" OR "theoretical sampl*" OR glaser AND strauss OR "content analy*" OR "thematic analy*" OR narrative* OR "unstructured categor*" OR "structured categor*" OR "unstructured interview*" OR "semi-structured interview*" OR "maximum variation*" OR snowball OR audio* OR tape* OR video* OR metasynthes* OR "meta-synthes*" OR metasummar* OR "meta-summar*" OR metastud* OR "meta-stud*" OR "meta-ethnograph*" OR metaethnog* OR "meta-narrative*" OR metanarrat* OR " meta-interpretation*" OR metainterpret* OR "qualitative meta-analy*" OR "qualitative metaanaly*" OR "qualitative metanaly*" OR "purposive sampl*" OR "action research" OR "focus group*" or photovoice or "photo voice" or "mixed method*" ) AND |  |
| 3 | ( MW United Kingdom OR ( TI ( ("national health service*" or nhs*) ) OR AB ( ("national health service*" or nhs*) ) ) OR ( TI ( (gb or "g.b." or britain* or (british* not "british columbia") or uk or "u.k." or "united kingdom*" or (england* not "new england") or "northern ireland*" or "northern irish*" or scotland* or scottish* or ((wales or "south wales") not "new south wales") or welsh*) ) OR AB ( (gb or "g.b." or britain* or (british* not "british columbia") or uk or "u.k." or "united kingdom*" or (england* not "new england") or "northern ireland*" or "northern irish*" or scotland* or scottish* or ((wales or "south wales") not "new south wales") or welsh*) OR MW ( (gb or "g.b." or britain* or (british* not "british columbia") or uk or "u.k." or "united kingdom*" or (england* not "new england") or "northern ireland*" or "northern irish*" or scotland* or scottish* or ((wales or "south wales") not "new south wales") or welsh*) ) ) |  |
| 4 | S1 AND S2 AND S3. Publication Date: 20171201-20240431 | 142 |

*Database: British Nursing Index (BNI)*

| **#** | **Query** | **Results from 19 Mar 2024** |
| --- | --- | --- |
| 1 | ((mainsubject((Breastfeeding)) OR title((breastfeed* OR breast feed* OR breastfed* OR breast fed OR breastmilk OR breast milk OR expressed milk* OR chestfeed* OR bodyfeed* OR chest fed OR body fed)) OR abstract((breastfeed* OR breast feed* OR breastfed* OR breast fed OR breastmilk OR breast milk OR expressed milk* OR chestfeed* OR bodyfeed* OR chest fed OR body fed)) OR title(infant* NEAR/2 feed*) OR title(nursing* AND (mother* OR birthing person OR birthing people* OR parent*))) AND noft((interview* OR experience* OR focus group* OR qualitative)) AND (location(United Kingdom) OR (title(((("national health service*" OR nhs* OR gb OR "g.b." OR britain* OR uk OR "u.k." OR "united kingdom*" OR england* OR "northern ireland*" OR "northern irish*" OR scotland* OR scottish* OR wales OR welsh*)))) OR abstract((("national health service*" OR nhs* OR gb OR "g.b." OR britain* OR uk OR "u.k." OR "united kingdom*" OR england* OR "northern ireland*" OR "northern irish*" OR scotland* OR scottish* OR wales OR welsh*)))))) AND pd(20171201-20240401) | 87 |

*Database: Scopus*

| **#** | **Query** |
| --- | --- |
| 1 | ( TITLE-ABS-KEY ( breastfeed* OR "breast feed*" OR breastfed* OR "breast fed" OR breastmilk OR * "breast milk" OR "expressed milk*" OR chestfeed* OR "chest feed*" OR bodyfeed* OR "body feed*" OR "chest fed" OR "body fed" ) OR TITLE-ABS-KEY ( nursing W/2 ( baby OR infant* OR newborn* OR mother* OR parent* OR "birthing people" OR "birthing person*" ) ) OR TITLE-ABS-KEY ( lactation ) OR TITLE-ABS-KEY ( ( infant* OR baby OR babies OR newborn* ) n/3 ( milk OR fed OR feed* OR lactat* ) ) |
| 2 | AND ALL ( qualitative OR ethnol* OR ethnog* OR ethnonurs* OR emic OR etic OR leininger OR noblit OR "field note*" OR "field record*" OR fieldnote* OR "field stud*" OR "participant observ*" OR "participant observation*" OR hermaneutic* OR phenomenolog* OR "lived experience*" OR heidegger* OR husserl* OR "merleau-pont*" OR colaizzi OR giorgi OR ricoeur OR spiegelberg OR "van kaam" OR "van manen" OR "grounded theory" OR "constant compar*" OR "theoretical sampl*" OR glaser AND strauss OR "content analy*" OR "thematic analy*" OR narrative* OR "unstructured categor*" OR "structured categor*" OR "unstructured interview*" OR "semi-structured interview*" OR "maximum variation*" OR snowball OR audio* OR tape* OR video* OR metasynthes* OR "meta-synthes*" OR metasummar* OR "meta-summar*" OR metastud* OR "meta-stud*" OR "meta-ethnograph*" OR metaethnog* OR "meta-narrative*" OR metanarrat* OR " meta-interpretation*" OR metainterpret* OR "qualitative meta-analy*" OR "qualitative metaanaly*" OR "qualitative metanaly*" OR "purposive sampl*" OR "action research" OR "focus group*" OR photovoice OR "photo voice" OR "mixed method*") |
| 3 | AND TITLE-ABS-KEY ( gb OR "g.b." OR britain* OR uk OR "u.k." OR "united kingdom*" OR england* OR "northern ireland*" OR "northern irish*" OR scotland* OR scottish* OR wales OR "south wales" ) ) AND PUBYEAR > 2017 AND PUBYEAR < 2025 |

*Database: Applied Social Sciences Index & Abstracts (ASSIA)*

| **#** | **Query** | **Results from 19 Mar 2024** |
| --- | --- | --- |
| 1 | mainsubject((Breastfeeding)) OR title((breastfeed* OR breast feed* OR breastfed* OR breast fed OR breastmilk OR breast milk OR expressed milk* OR chestfeed* OR bodyfeed* OR chest fed OR body fed)) OR abstract((breastfeed* OR breast feed* OR breastfed* OR breast fed OR breastmilk OR breast milk OR expressed milk* OR chestfeed* OR bodyfeed* OR chest fed OR body fed)) OR title(infant* NEAR/2 feed*) OR title(nursing* AND (mother* OR birthing person OR birthing people* OR parent*)) | 4728 |
| 2 | (interview* or experience* or focus group* or qualitative) | 487978 |
| 3 | [S1] AND [S2] | 2201 |
| 4 | ([S1] AND [S2]) AND location.exact("United Kingdom--UK" OR "England" OR "Scotland" OR "Wales" OR "Northern Ireland" OR "Ireland" OR "UK" OR "United Kingdom" OR "Edinburgh Scotland") | 196 |
| 5 | title(((("national health service*" OR nhs* OR gb OR "g.b." OR britain* OR uk OR "u.k." OR "united kingdom*" OR england* OR "northern ireland*" OR "northern irish*" OR scotland* OR scottish* OR wales OR welsh*)))) OR abstract((("national health service*" OR nhs* OR gb OR "g.b." OR britain* OR uk OR "u.k." OR "united kingdom*" OR england* OR "northern ireland*" OR "northern irish*" OR scotland* OR scottish* OR wales OR welsh*))) | 94586 |
| 6 | [S3] AND [S5] | 224 |
| 7 | [S4] OR [S6] | 313 |
| 8 | ([S4] OR [S6]) AND pd(20171201-20240401) | 104 |

*Database: Social Policy and Practice <202402>*

| **#** | **Query** | **Results from 20 Mar 2024** |
| --- | --- | --- |
| 1 | (breastfeed* or breast feed* or breastfed* or breast fed or breastmilk or breast milk or expressed milk* or chestfeed* or chest feed* or bodyfeed* body feed* or chest fed or body fed).af. | 255 |
| 2 | (nursing adj2 (baby or infant* or newborn* or mother* or parent* or birthing people or birthing person*)).af. | 25 |
| 3 | ((infant* or baby or babies or newborn*) adj3 (milk or fed or feed* or lactat*)).af. | 54 |
| 4 | lactation.af. | 3 |
| 5 | 1 or 2 or 3 or 4 | 307 |
| 6 | limit 5 to yr="2017 -Current" | 42 |
