# Supplementary material for: Peer Support and Community Interventions Targeting Breastfeeding in the UK: Systematic Review of Qualitative Evidence to Identify Inequities in Participants' Experiences
Source: Matern Child Nutr. 2025 May 19;21(4):e70041. doi: 10.1111/mcn.70041 (PMC12454195; doi:10.1111/mcn.70041)
Supplement: Supplementary file 3 — Supplement B. [file MCN-21-e70041-s003.docx]

**Supplement B**

*Review eligibility criteria: Developed in accordance with SPIDER*

| **SPIDER Domains** | **Inclusion Criteria** |
| --- | --- |
| Sample | Women and birthing people who have current or prior experience of breastfeeding or chest feeding, may breastfeed or chest feed in future, or have not been able to breastfeed or chest feed. No age restrictions.  Stakeholders involved with the development, delivery and/or funding of interventions (e.g. peer supporters). |
| Phenomena of Interest | Intervention participants’ experiences and/or views of intervention. May relate to reach and recruitment; retention; interaction; and sustainment. Non-participating stakeholders must provide reflections on the experiences and/or views of participants. |
| Design | Any study design using a research method collecting experiences and/or views of individuals defined by the sample. |
| Evaluation | Breastfeeding interventions:   1. Peer-led support: Support offered by trained women or birthing people who have themselves breastfed or chest fed. May have the same socio-economic background, ethnicity, or locality as the individuals they are supporting. May also include women and birthing people offering peer support without formal training but in an organised manner (e.g. dedicated Facebook or WhatsApp communities). Can be delivered in any setting. 2. Community intervention: Non-hospital-based support provided through local and regional community networks. May be facilitated via professional health care agencies (e.g. community health visitors) or non-health care professionals.   Interventions may operate through a number of mechanisms (e.g. increased parent-child bonding or knowledge development).  Intervention outcomes must be specified as breastfeeding or chest feeding initiation, maintenance and/or (early) cessation. |
| Research Type | Any type of qualitative research. |
| Country | UK; England; Northern Ireland; Scotland; Wales |
| Years | All. Some elements of the search employed date limits as the purpose was to update an existing systematic review. This is detailed in the manuscripts methods section. |
