# Supplementary material for: Peer Support and Community Interventions Targeting Breastfeeding in the UK: Systematic Review of Qualitative Evidence to Identify Inequities in Participants' Experiences
Source: Matern Child Nutr. 2025 May 19;21(4):e70041. doi: 10.1111/mcn.70041 (PMC12454195; doi:10.1111/mcn.70041)
Supplement: Supplementary file 4 — Supplement C. [file MCN-21-e70041-s002.docx]

**Supplement C**

*Study reports for each phase of intervention participation*

| **Intervention Phase** | **Studies (n)** | **Study Reports** |
| --- | --- | --- |
| Reach and recruitment | 17 | (Aiken & Thomson, 2013); (Beake et al., 2005); (Brown et al., 2021); (Brown & Tennant-Eyles, 2021); (Carrington-Windo, 2018); (Clarke, Ingram, Johnson, Thomson, Trickey, & Dombrowski, 2020); (Clarke, Ingram, Johnson, Thomson, Trickey, Dombrowski, et al., 2020); (Cook et al., 2021); (Fox et al., 2015); (Fraser et al., 2020); (Hunt, 2019); (Hunt et al., 2021); (Ingram et al., 2020); (Islam, 2016); (Jackson & Hallam, 2019), (Jackson & Hallam, 2021)); (Knox et al., 2023); (McFadden et al., 2013); (Mengoni et al., 2023); (Simmons, 2021); (Thomson, Crossland, et al., 2012); (Thomson & Crossland, 2013); (Thomson et al., 2015); (Thomson et al., 2020); (Thomson et al., 2022). |
| Retention and drop out | 4 | (Brown & Tennant-Eyles, 2021); (Clarke, Ingram, Johnson, Thomson, Trickey, & Dombrowski, 2020); (Clarke, Ingram, Johnson, Thomson, Trickey, Dombrowski, et al., 2020); (Ingram et al., 2020); (Jackson & Hallam, 2019); (Jackson & Hallam, 2021); (Knox et al., 2023); (Thomson et al., 2020); (Thomson et al., 2022); (Trickey, 2018). |
| Interaction | 54 | (Beake et al., 2005); (Brown et al., 2021); (Carrington-Windo, 2018); (Cartwright & Boath, 2018); (Condon & Salmon, 2015); (Cook et al., 2021); (Crossland et al., 2019); (Crossland et al., 2020); (Dombrowski et al., 2020); (Fraser et al., 2020); (Graffy & Taylor, 2005); (Hoddinott et al., 2012); (Jackson et al., 2023); (Jamie et al., 2020); (Johnson et al., 2018); (Miller, 2022); (H. Morse & A. Brown, 2022); (Newman & Williamson, 2018); (Simmons, 2021); (Smyth, 2020), (Aiken & Thomson, 2013); (Battersby, 2001); (Black et al., 2020); (Brown & Tennant-Eyles, 2021); (Clarke, Ingram, Johnson, Thomson, Trickey, & Dombrowski, 2020); (Clarke, Ingram, Johnson, Thomson, Trickey, Dombrowski, et al., 2020); (Copeland et al., 2019); (Dowling & Pontin, 2017); (Hoddinott et al., 2006); (Hunt & Thomson, 2017); (Hunt, 2019); (Hunt et al., 2021); (Ingram et al., 2005); (Ingram, 2013); (Ingram et al., 2020); (Knox et al., 2023); (Lyons et al., 2019); (Mengoni et al., 2023); (Paranjothy et al., 2017); (Phillips et al., 2018); (Regan & Brown, 2019); (Scott et al., 2003); (Thomson, Crossland, et al., 2012); (Thomson, Dykes, et al., 2012); (Thomson & Crossland, 2013); (Thomson et al., 2015); (Thomson & Crossland, 2019); (Thomson et al., 2020); (Thomson et al., 2022) (Trickey, 2018); (Wade et al., 2009); (Fox et al., 2015); (Gallagher, 2023); (Ingram et al., 2008); (Jackson & Hallam, 2019); (Jackson & Hallam, 2021); (Marshall et al., 2007); (McFadden & Toole, 2006); (McFadden et al., 2013); (Morse & Brown, 2021); (Holly Morse & Amy Brown, 2022); (Psarros, 2018); (Raine, 2003); (Tan et al., 2017); (Thompson et al., 2020); (Wagg et al., 2021); (Wagg et al., 2022). |
| Sustainment | 7 | (Brown et al., 2021); (Copeland et al., 2019); (Phillips et al., 2018); (Paranjothy et al., 2017); (Hunt, 2019); (Hunt et al., 2021); (Ingram, 2013); (Jackson & Hallam, 2019); (Jackson & Hallam, 2021); (Thomson, Crossland, et al., 2012); (Thomson et al., 2015); (Wade et al., 2009). |

**References**

Aiken, A., & Thomson, G. (2013). Professionalisation of a breast-feeding peer support service: issues and experiences of peer supporters. *Midwifery*, *29*(12), e145-151. <https://doi.org/10.1016/j.midw.2012.12.014>

Battersby, S. (2001). The Worldly Wise project. A different approach to breastfeeding support. *Pract Midwife*, *4*(6), 30-31. <https://www.ncbi.nlm.nih.gov/pubmed/12026844>

Beake, S., McCourt, C., Rowan, C., & Taylor, J. (2005). Evaluation of the use of health care assistants to support disadvantaged women breastfeeding in the community. *Matern Child Nutr*, *1*(1), 32-43. <https://doi.org/10.1111/j.1740-8709.2004.00007.x>

Black, R., McLaughlin, M., & Giles, M. (2020). Women's experience of social media breastfeeding support and its impact on extended breastfeeding success: A social cognitive perspective. *British journal of health psychology*, *25*(3), 754-771. <https://doi.org/https://dx.doi.org/10.1111/bjhp.12451>

Brown, A., Jones, S., Grant, A., Coffey, M., & Mayers, A. (2021). An Evaluation of the Peppy Baby Programme in Manchester University Foundation Trust. <https://www.england.nhs.uk/north-west/wp-content/uploads/sites/48/2021/10/An-Evaluation-of-the-Peppy-Baby-Programme-in-Manchester-University-Foundation-Trust.pdf>

Brown, R., & Tennant-Eyles, A. (2021). *The Breastfeeding Network. An exploration of trends and experiences of delivery of breastfeeding peer support in England and Wales, since 2015*. <https://www.breastfeedingnetwork.org.uk/wp-content/uploads/2022/06/BFN-Cardiff-Report-27.06.22.pdf>

Carrington-Windo, A. (2018). *Successful breastfeeding? : investigating mothers' experiences of infant feeding policies in the United Kingdom* University of Oxford (United Kingdom)]. England. [https://uoelibrary.idm.oclc.org/login?url=https://www.proquest.com/dissertations-theses/successful-breastfeeding-investigating-mothers/docview/2372446857/se-2?accountid=10792https://libkey.io/libraries/394/openurl?genre=dissertations&au=Carrington-Windo%2](https://uoelibrary.idm.oclc.org/login?url=https://www.proquest.com/dissertations-theses/successful-breastfeeding-investigating-mothers/docview/2372446857/se-2?accountid=10792https://libkey.io/libraries/394/openurl?genre=dissertations&au=Carrington-Windo%252)

Cartwright, A., & Boath, E. (2018). Feeding infants with Down's Syndrome: A qualitative study of mothers' experiences. *Journal of Neonatal Nursing*, *24*(3), 134-141. <https://doi.org/10.1016/j.jnn.2018.03.001>

Clarke, J. L., Ingram, J., Johnson, D., Thomson, G., Trickey, H., & Dombrowski, S. U. (2020). An assets-based intervention before and after birth to improve breastfeeding initiation and continuation: the ABA feasibility RCT. *Public Health Research*, *8*(7).

Clarke, J. L., Ingram, J., Johnson, D., Thomson, G., Trickey, H., Dombrowski, S. U., Sitch, A., Dykes, F., Feltham, M., MacArthur, C., Roberts, T., Hoddinott, P., & Jolly, K. (2020). The ABA intervention for improving breastfeeding initiation and continuation: Feasibility study results. *Matern Child Nutr*, *16*(1), e12907. <https://doi.org/10.1111/mcn.12907>

Condon, L. J., & Salmon, D. (2015). 'You likes your way, we got our own way': Gypsies and Travellers' views on infant feeding and health professional support. *Health Expect*, *18*(5), 784-795. <https://doi.org/10.1111/hex.12214>

Cook, E. J., Powell, F., Ali, N., Penn-Jones, C., Ochieng, B., & Randhawa, G. (2021). Improving support for breastfeeding mothers: a qualitative study on the experiences of breastfeeding among mothers who reside in a deprived and culturally diverse community. *International journal for equity in health*, *20*(1), 92. <https://doi.org/https://dx.doi.org/10.1186/s12939-021-01419-0>

Copeland, L., Merrett, L., McQuire, C., Grant, A., Gobat, N., Tedstone, S., Playle, R., Channon, S., Sanders, J., Phillips, R., Hunter, B., Brown, A., Fitzsimmons, D., Robling, M., & Paranjothy, S. (2019). Feasibility and acceptability of a motivational interviewing breastfeeding peer support intervention. *Matern Child Nutr*, *15*(2), e12703. <https://doi.org/10.1111/mcn.12703>

Crossland, N., Thomson, G., & Moran, V. H. (2019). Embedding supportive parenting resources into maternity and early years care pathways: a mixed methods evaluation. *BMC pregnancy and childbirth*, *19*(1), 253. <https://doi.org/https://dx.doi.org/10.1186/s12884-019-2388-2>

Crossland, N., Thomson, G., & Moran, V. H. (2020). Impact of parenting resources on breastfeeding, parenting confidence and relationships. *Midwifery*, *81*, 102591. <https://doi.org/https://dx.doi.org/10.1016/j.midw.2019.102591>

Dombrowski, L., Henderson, S., Leslie, J., Mohammed, K., Johnson, D., & Allan, N. (2020). The role of early years care providers in supporting continued breastfeeding and breast milk feeding. *Early Years: An International Journal of Research and Development*, *40*(2), 205-220. <https://doi.org/https://dx.doi.org/10.1080/09575146.2018.1430123>

Dowling, S., & Pontin, D. (2017). Using liminality to understand mothers' experiences of long-term breastfeeding: 'Betwixt and between', and 'matter out of place'. *Health (London, England : 1997)*, *21*(1), 57-75. <https://doi.org/https://dx.doi.org/10.1177/1363459315595846>

Fox, R., McMullen, S., & Newburn, M. (2015). UK women's experiences of breastfeeding and additional breastfeeding support: a qualitative study of Baby Cafe services. *BMC Pregnancy Childbirth*, *15*(1), 147. <https://doi.org/10.1186/s12884-015-0581-5>

Fraser, M., Dowling, S., Oxford, L., Ellis, N., & Jones, M. (2020). Important times for breastfeeding support: a qualitative study of mothers' experiences. *International Journal of Health Promotion and Education*, *58*(2), 71-82. <https://doi.org/https://dx.doi.org/10.1080/14635240.2019.1676811>

Gallagher, J. (2023). A biographical narrative exploration of infant feeding in an area with low breastfeeding rates. *Dissertation Abstracts International: Section B: The Sciences and Engineering*, *84*(12-B), No-Specified. <http://ovidsp.ovid.com/ovidweb.cgi?T=JS&PAGE=reference&D=psyc22&NEWS=N&AN=2023-93852-170>

Graffy, J., & Taylor, J. (2005). What information, advice, and support do women want with breastfeeding? *Birth*, *32*(3), 179-186. <https://doi.org/10.1111/j.0730-7659.2005.00367.x>

Hoddinott, P., Chalmers, M., & Pill, R. (2006). One-to-one or group-based peer support for breastfeeding? Women's perceptions of a breastfeeding peer coaching intervention. *Birth*, *33*(2), 139-146. <https://doi.org/10.1111/j.0730-7659.2006.00092.x>

Hoddinott, P., Craig, L. C. A., Britten, J., & McInnes, R. M. (2012). A serial qualitative interview study of infant feeding experiences: Idealism meets realism. *BMJ Open*, *2*(2). <https://doi.org/10.1136/bmjopen-2011-000504>

Hunt, L., & Thomson, G. (2017). Pressure and judgement within a dichotomous landscape of infant feeding: a grounded theory study to explore why breastfeeding women do not access peer support provision. *Matern Child Nutr*, *13*(2). <https://doi.org/10.1111/mcn.12279>

Hunt, L., Thomson, G., Whittaker, K., & Dykes, F. (2021). Adapting breastfeeding support in areas of socio-economic deprivation: a case study approach. *Int J Equity Health*, *20*(1), 83. <https://doi.org/10.1186/s12939-021-01393-7>

Hunt, L. S. (2019). *An Exploration of How Third Sector Breastfeeding Support Organisations Have Developed Their Services for Delivery in Areas of Socio-Economic Deprivation* University of Central Lancashire (United Kingdom)]. England. <https://uoelibrary.idm.oclc.org/login?url=https://www.proquest.com/dissertations-theses/exploration-how-third-sector-breastfeeding/docview/2475184133/se-2?accountid=10792https://libkey.io/libraries/394/openurl?genre=dissertations&au=Hunt%2C+Louise+Susan&a>

Ingram, J. (2013). A mixed methods evaluation of peer support in Bristol, UK: mothers', midwives' and peer supporters' views and the effects on breastfeeding. *BMC Pregnancy Childbirth*, *13*, 192. <https://doi.org/10.1186/1471-2393-13-192>

Ingram, J., Cann, K., Peacock, J., & Potter, B. (2008). Exploring the barriers to exclusive breastfeeding in black and minority ethnic groups and young mothers in the UK. *Matern. Child Nutr.*, *4*(3), 171-180. <https://doi.org/10.1111/j.1740-8709.2007.00129.x>

Ingram, J., Rosser, J., & Jackson, D. (2005). Breastfeeding peer supporters and a community support group: evaluating their effectiveness. *Matern Child Nutr*, *1*(2), 111-118. <https://doi.org/10.1111/j.1740-8709.2005.00005.x>

Ingram, J., Thomson, G., Johnson, D., Clarke, J. L., Trickey, H., Hoddinott, P., Dombrowski, S. U., Jolly, K., & Team, A. B. A. S. (2020). Women's and peer supporters' experiences of an assets-based peer support intervention for increasing breastfeeding initiation and continuation: A qualitative study. *Health Expect*, *23*(3), 622-631. <https://doi.org/10.1111/hex.13042>

Islam, M. P. (2016). Why are 'hard-to-reach' women not engaging in a breastfeeding peer support programme? *Community Pract*, *89*(2), 36-41. <https://www.ncbi.nlm.nih.gov/pubmed/27164801>

Jackson, J. E., & Hallam, J. (2019). ‘I felt like I was doing something wrong’: A qualitative exploration of mothers' experiences of breastfeeding. *Journal of Health Visiting*, *7*(4), 166-172. <https://doi.org/https://doi.org/10.12968/johv.2019.7.4.166>

Jackson, J. E., & Hallam, J. (2021). Against all odds-why UK mothers' breastfeeding beyond infancy are turning to their international peers for emotional and informative support. *Health care for women international*, *42*(4-6), 739-755. <https://doi.org/https://dx.doi.org/10.1080/07399332.2020.1744147>

Jackson, J. E., Wild, R., Hallam, J., Graves, R., Woodstein, B. J., & Stothard, P. (2023). Exploring the healthcare experiences and support needs of chestfeeding or breastfeeding for trans and non-binary parents based in the united kingdom. *International Journal of Transgender Health*, No-Specified. <https://doi.org/https://dx.doi.org/10.1080/26895269.2023.2265371>

Jamie, K., O'Neill, R., Bows, H., & Hackshaw-McGeagh, L. (2020). Healthcare practitioner relationships, cultural health capital and breastfeeding support for adolescent mothers. *Health Education Journal*, *79*(8), 901-913. <https://doi.org/https://dx.doi.org/10.1177/0017896920915945>

Johnson, M., Whelan, B., Relton, C., Thomas, K., Strong, M., Scott, E., & Renfrew, M. J. (2018). Valuing breastfeeding: a qualitative study of women's experiences of a financial incentive scheme for breastfeeding. *BMC pregnancy and childbirth*, *18*(1), 20. <https://doi.org/https://dx.doi.org/10.1186/s12884-017-1651-7>

Knox, O., Parker, D., Johnson, D., Dombrowski, S. U., Thomson, G., Clarke, J., Hoddinott, P., Jolly, K., & Ingram, J. (2023). Text message conversations between peer supporters and women to deliver infant feeding support using behaviour change techniques: A qualitative analysis. *Midwifery*, *127*, 103838. <https://doi.org/https://dx.doi.org/10.1016/j.midw.2023.103838>

Lyons, S., Currie, S., & Smith, D. M. (2019). Learning from Women with a Body Mass Index (Bmi) >= 30 kg/m2 who have Breastfed and/or are Breastfeeding: a Qualitative Interview Study. *Maternal and child health journal*, *23*(5), 648-656. <https://doi.org/https://dx.doi.org/10.1007/s10995-018-2679-7>

Marshall, J. L., Godfrey, M., & Renfrew, M. J. (2007). Being a 'good mother': managing breastfeeding and merging identities. *Soc Sci Med*, *65*(10), 2147-2159. <https://doi.org/10.1016/j.socscimed.2007.06.015>

McFadden, A., Renfrew, M. J., & Atkin, K. (2013). Does cultural context make a difference to women's experiences of maternity care? A qualitative study comparing the perspectives of breast-feeding women of Bangladeshi origin and health practitioners. *Health Expect*, *16*(4), e124-135. <https://doi.org/10.1111/j.1369-7625.2012.00770.x>

McFadden, A., & Toole, G. (2006). Exploring women's views of breastfeeding: a focus group study within an area with high levels of socio-economic deprivation. *Matern Child Nutr*, *2*(3), 156-168. <https://doi.org/10.1111/j.1740-8709.2006.00054.x>

Mengoni, S. E., Smith, B., Wythe, H., & Rogers, S. L. (2023). Experiences of feeding young children with Down syndrome: parents' and health professionals' perspectives. *International Journal of Developmental Disabilities*. <https://doi.org/https://dx.doi.org/10.1080/20473869.2023.2269321>

Miller, A. (2022). *A Mixed-Methods Study of Student Experiences and Mother-Baby Outcomes in a Novel Interprofessional Student-Led Breastfeeding Clinic* Bournemouth University (United Kingdom)]. England. <https://uoelibrary.idm.oclc.org/login?url=https://www.proquest.com/dissertations-theses/mixed-methods-study-student-experiences-mother/docview/2796601822/se-2?accountid=10792https://libkey.io/libraries/394/openurl?genre=dissertations&au=Miller%2C+Amy&aula>

Morse, H., & Brown, A. (2021). Accessing local support online: Mothers' experiences of local Breastfeeding Support Facebook groups. *Maternal & child nutrition*, *17*(4), e13227. <https://doi.org/https://dx.doi.org/10.1111/mcn.13227>

Morse, H., & Brown, A. (2022). Mothers' experiences of using Facebook groups for local breastfeeding support: Results of an online survey exploring midwife moderation. *PLOS digital health*, *1*(11), e0000144. <https://doi.org/https://dx.doi.org/10.1371/journal.pdig.0000144>

Morse, H., & Brown, A. (2022). "Running on goodwill and fairydust" - midwives' experiences of facilitating and delivering local breastfeeding support via Facebook groups: a qualitative descriptive study. *medRxiv*. <https://doi.org/https://dx.doi.org/10.1101/2022.10.18.22281224>

Newman, K. L., & Williamson, I. R. (2018). Why aren't you stopping now?!' Exploring accounts of white women breastfeeding beyond six months in the East of England. *Appetite*, *129*, 228-235. <https://doi.org/https://dx.doi.org/10.1016/j.appet.2018.06.018>

Paranjothy, S., Copeland, L., Merrett, L., Grant, A., Phillips, R., Gobat, N., Sanders, J., Fitzsimmons, D., Hunter, B., Regan, S., Playle, R., Brown, A., Tedstone, S., Trickey, H., & Robling, M. (2017). A novel peer-support intervention using motivational interviewing for breastfeeding maintenance: a UK feasibility study. *Health technology assessment (Winchester, England)*, *21*(77), 1-138. <https://doi.org/https://dx.doi.org/10.3310/hta21770>

Phillips, R., Copeland, L., Grant, A., Sanders, J., Gobat, N., Tedstone, S., Stanton, H., Merrett, L., Rollnick, S., Robling, M., Brown, A., Hunter, B., Fitzsimmons, D., Regan, S., Trickey, H., & Paranjothy, S. (2018). Development of a novel motivational interviewing (MI) informed peer-support intervention to support mothers to breastfeed for longer. *BMC Pregnancy Childbirth*, *18*(1), 90. <https://doi.org/10.1186/s12884-018-1725-1>

Psarros, A. (2018). *Mothers' Voices: Exploring experiences of maternity and health in low income women and children from diverse ethnic backgrounds*. <https://maternityaction.org.uk/wp-content/uploads/MothersVoices2018-FINAL.pdf>

Raine, P. (2003). Promoting breast-feeding in a deprived area: the influence of a peer support initiative. *Health Soc Care Community*, *11*(6), 463-469. <https://doi.org/10.1046/j.1365-2524.2003.00449.x>

Regan, S., & Brown, A. (2019). Experiences of online breastfeeding support: Support and reassurance versus judgement and misinformation. *Maternal & child nutrition*, *15*(4), e12874. <https://doi.org/https://dx.doi.org/10.1111/mcn.12874>

Scott, J. A., Mostyn, T., & Greater Glasgow Breastfeeding Initiative Management, T. (2003). Women's experiences of breastfeeding in a bottle-feeding culture. *J Hum Lact*, *19*(3), 270-277. <https://doi.org/10.1177/0890334403255225>

Simmons, H. (2021). Surveillance of modern motherhood: An exploration of the experiences of mothers that have attended a universal parenting course. *Dissertation Abstracts International Section A: Humanities and Social Sciences*, *82*(10-A), No-Specified. <http://ovidsp.ovid.com/ovidweb.cgi?T=JS&PAGE=reference&D=psyc20&NEWS=N&AN=2021-50283-016>

Smyth, L. (2020). Social roles and alienation: Breastfeeding promotion and early motherhood. *Current Sociology*, *68*(6), 814-831. <https://doi.org/10.1177/0011392118807512>

Tan, M., Rheeston, M., & Douglas, H. (2017). Using the Solihull Approach in breastfeeding support groups: Maternal perceptions. *British Journal of Midwifery*, *25*(12), 765-773. <https://doi.org/10.12968/bjom.2017.25.12.765>

Thompson, A. J., Topping, A. E., & Jones, L. L. (2020). 'Surely you're not still breastfeeding': a qualitative exploration of women's experiences of breastfeeding beyond infancy in the UK. *BMJ Open*, *10*(5), e035199. <https://doi.org/https://dx.doi.org/10.1136/bmjopen-2019-035199>

Thomson, G., Balaam, M. C., & Hymers, K. (2015). Building social capital through breastfeeding peer support: insights from an evaluation of a voluntary breastfeeding peer support service in North-West England. *Int Breastfeed J*, *10*(1), 15. <https://doi.org/10.1186/s13006-015-0039-4>

Thomson, G., & Crossland, N. (2013). Callers' attitudes and experiences of UK breastfeeding helpline support. *Int Breastfeed J*, *8*(1), 3. <https://doi.org/10.1186/1746-4358-8-3>

Thomson, G., & Crossland, N. (2019). Using the behaviour change wheel to explore infant feeding peer support provision; insights from a North West UK evaluation. *Int Breastfeed J*, *14*(1), 41. <https://doi.org/10.1186/s13006-019-0236-7>

Thomson, G., Crossland, N., & Dykes, F. (2012). Giving me hope: women's reflections on a breastfeeding peer support service. *Matern Child Nutr*, *8*(3), 340-353. <https://doi.org/10.1111/j.1740-8709.2011.00358.x>

Thomson, G., Dykes, F., Hurley, M. A., & Hoddinott, P. (2012). Incentives as connectors: insights into a breastfeeding incentive intervention in a disadvantaged area of North-West England. *BMC Pregnancy Childbirth*, *12*, 22. <https://doi.org/10.1186/1471-2393-12-22>

Thomson, G., Ingram, J., Clarke, J., Johnson, D., & Jolly, K. (2022). Who Gets to Breastfeed? A Narrative Ecological Analysis of Women's Infant Feeding Experiences in the UK. *Frontiers in sociology*, *7*, 904773. <https://doi.org/https://dx.doi.org/10.3389/fsoc.2022.904773>

Thomson, G., Ingram, J., Clarke, J. L., Johnson, D., Trickey, H., Dombrowski, S. U., Hoddinott, P., Darwent, K., & Jolly, K. (2020). Exploring the use and experience of an infant feeding genogram to facilitate an assets-based approach to support infant feeding. *BMC pregnancy and childbirth*, *20*(1), 569. <https://doi.org/https://dx.doi.org/10.1186/s12884-020-03245-8>

Trickey, H. (2018). *Theorising Breastfeeding Peer Support as Intervention in a Complex Ecological System : Lessons for Implementation and Evaluation in a Welsh Context* Cardiff University (United Kingdom)]. Wales. <https://uoelibrary.idm.oclc.org/login?url=https://www.proquest.com/dissertations-theses/theorising-breastfeeding-peer-support-as/docview/2593858606/se-2?accountid=10792https://libkey.io/libraries/394/openurl?genre=dissertations&au=Trickey%2C+Heather&aulas>

Wade, D., Haining, S., & Day, A. (2009). Breastfeeding peer support: are there additional benefits? *Community practitioner : the journal of the Community Practitioners' & Health Visitors' Association*, *82*(12), 30-33.

Wagg, A. J., Callanan, M. M., & Hassett, A. (2021). Exploring Online Breastfeeding Support Groups, Part 1 of 2: Finding a Community of Like-Minded People Helps a Mother to Reach Her Breastfeeding Goals. *Clinical Lactation*, *12*(4), 184-192. <https://doi.org/10.1891/cl-2021-0008>

Wagg, A. J., Hassett, A., & Callanan, M. M. (2022). Exploring Online Social Support Groups, Part 2: "There's Just Pictures on Their Everyday and That's the Only Thing That Normalizes It for Me". *Clinical Lactation*, *13*(1), 24-31. <https://doi.org/10.1891/CL.2021-0014>
